# Alteration of CTCF-associated chromatin neighborhood inhibits *TAL1*-driven oncogenic transcription program and leukemogenesis

**DOI:** 10.1093/nar/gkaa098

**Published:** 2020-02-22

**Authors:** Ying Li, Ziwei Liao, Huacheng Luo, Aissa Benyoucef, Yuanyuan Kang, Qian Lai, Sinisa Dovat, Barbara Miller, Iouri Chepelev, Yangqiu Li, Keji Zhao, Marjorie Brand, Suming Huang

**Affiliations:** 1 Department of Pediatrics and Pharmacology, Pennsylvania State University College of Medicine, Hershey, PA 17033, USA; 2 Department of Biochemistry & Molecular Biology, University of Florida College of Medicine, Gainesville, FL 32610, USA; 3 Institute of Hematology, Jinan University Medical College, ShiPai, Guangzhou, 510632, China; 4 The Sprott Center for Stem Cell Research, Regenerative Medicine Program, Ottawa Hospital Research Institute, Ottawa, Ontario K1H 8L6, Canada; 5 Laboratory of Molecular Immunology, National Heart, Lung and Blood Institute, NIH, Bethesda, MD 20814, USA; 6 Center for Autoimmune Genomics and Etiology, Cincinnati Children's Hospital Medical Center, Cincinnati, OH 45229, USA

## Abstract

Aberrant activation of the *TAL1* is associated with up to 60% of T-ALL cases and is involved in CTCF-mediated genome organization within the *TAL1* locus, suggesting that CTCF boundary plays a pathogenic role in T-ALL. Here, we show that −31-Kb CTCF binding site (*−31CBS*) serves as chromatin boundary that defines topologically associating domain (TAD) and enhancer/promoter interaction required for *TAL1* activation. Deleted or inverted *−31CBS* impairs *TAL1* expression in a context-dependent manner. Deletion of *−31CBS* reduces chromatin accessibility and blocks long-range interaction between the +51 erythroid enhancer and *TAL1 promoter-1* leading to inhibition of *TAL1* expression in erythroid cells, but not T-ALL cells. However, in *TAL1-*expressing T-ALL cells, the leukemia-prone *TAL1 promoter-IV* specifically interacts with the *+19* stem cell enhancer located 19 Kb downstream of *TAL1* and this interaction is disrupted by the *−31CBS* inversion in T-ALL cells. Inversion of *−31CBS* in Jurkat cells alters chromatin accessibility, histone modifications and CTCF-mediated TAD leading to inhibition of *TAL1* expression and TAL1-driven leukemogenesis. Thus, our data reveal that *−31CBS acts as critical regulator to define +19-*enhancer and the leukemic prone promoter IV interaction for *TAL1* activation in T-ALL. Manipulation of CTCF boundary can alter *TAL1* TAD and oncogenic transcription networks in leukemogenesis.

## INTRODUCTION

T-cell acute lymphoblastic leukemia (T-ALL) is an aggressive fatal disease that affects both children and adults. Approximately 30% of T-ALL cases relapse within the first 2 years following diagnosis (1–4). The poor prognosis is a consequence of insufficient knowledge of molecular mechanisms underlying T-ALL pathogenesis. Better understanding of the molecular changes associated with T-ALL biology will lead to development of novel diagnostic and therapeutic strategies.

Activation of TAL1, a basic helix-loop-helix (bHLH) transcription factor, is the most frequent gain-of-function mutation observed in T-ALL patients and is found in 40–60% of T-ALL cases resulted from chromosomal translocation (4–5%), interstitial chromosome deletion (25–30%), or an undefined mechanism (60%) (5–7). Upregulation of *Tal1* in T-cells also led to leukemia in mice (8,9). Deletion of *TAL1* in T-ALL eliminated the leukemic phenotype and induced apoptosis (10–12), implicating one important role of *TAL1* in T-cell neoplastic disease. Despite having identified several enhancers in *TAL1* regulation, it remains largely unknown how these enhancers are differentially utilized and whether they are involved in *TAL1* aberration in T-ALL.

TAL1 is required for HSC self-renewal (13) and the commitment of hematopoietic lineages (14,15). Deletion of *Tal1* in mice leads to embryonic lethality at embryonic day 9.5 (E9.5) due to a complete loss of hematopoietic cells (16,17). Further, *Tal1*-null embryonic stem cells (ESCs) are unable to generate both primitive and definitive erythroid cells *in vitro* and do not contribute to hematopoiesis *in vivo* in a chimeric mouse (18,19). These results demonstrate that TAL1 acts as a master regulator of hematopoiesis. Because of its relevance to normal hematopoiesis and T-ALL, transcriptional regulation of the *TAL1* becomes a fundamental issue for controlling normal and malignant hematopoiesis.

The human *TAL1* gene is located on chromosome 1p32 and is tightly regulated by various *cis* regulatory elements (20–23). The organization of this 199-Kb gene dense region are conserved among chicken, mouse, and human genomes (21). Several studies including transgenic reporter and knock-in mouse, DNase I hypersensitive assay, and ChIP on chip assays have revealed that expression of the *TAL1* during hematopoiesis is controlled by distinct promoters and enhancers. Some of these enhancers are located far away from the transcription start site (TSS) of the *TAL1* gene (23–26,20). However, the detailed mechanisms governing differential enhancer/promoter actions that selectively activate *TAL1* in different stages of hematopoiesis and leukemogenesis remain unclear and must still be illustrated.

Genome-wide studies of K562 cells (27) and CD4^+^ T cells (28) revealed that there are four CTCF binding sites (CBSs) in the *TAL1* locus bound by CTCF to separate the *STIL*, *TAL1* and *MAP17* genes (27,29). CTCF, as enhancer-blocking insulator, prevents enhancer and promoter interactions when placed between them. CTCF also acts as chromatin boundary to play a critical role in defining topologically associating domains (TAD) and chromatin signature within the TAD (30,31). CTCF is a highly conserved zinc-finger protein involved in transcription activation/repression, insulation, imprinting and X chromosome inactivation (32–35). Recent studies implicated that CTCF regulates intra- and interchromosomal contacts within the nucleus at several developmentally regulated genomic loci (36,37) and suggested a primary function for CTCF in global organization of chromatin architecture (32,38). It is conceivable that altered CTCF defined boundary might result in inappropriate enhancer/promoter interactions leading to changes in transcription of oncogene or tumor suppressor. We and others showed that the −31Kb CBS *(−31CBS*) plays a critical role in organizing enhancer/promoter communications to activate *TAL1* (39,40). However, it remains to be determined whether CTCF is directly involved in enhancer/promoter interactions for *TAL1* activation. Furthermore, whether and how aberrant *TAL1* activation is depended on CTCF defined chromatin neighborhood within the *TAL1* locus. Defining molecular mechanisms that are involved in differential activation of *TAL1* are critical for understanding its role in the pathogenesis of T-ALL for potential target therapy.

Here, we demonstrated that inversion of the *−31CBS* orientation alters *TAL1* three-dimensional genome organization and chromatin signature in the *TAL1* locus that results in inhibition of the TAL1-driven oncogenic transcription program and T-cell leukemogenesis. Thus, targeting the CTCF-mediated chromatin neighborhood provides an opportunity to correct the aberrant oncogene transcription program and to develop new molecular therapy for acute leukemia.

## MATERIALS AND METHODS

### Patient samples and cell lines

Primary T-ALL patient samples including TAL1-positive (08H125) and TAL1-negative (08H028) blasts were obtained from the Quebec Leukemia Cell Bank (41) and expanded in non-obese diabetic (NOD)/LtSz-severe combined immunodeficiency (SCID) IL2Rγ_c_^null^ (NSG) female mice as previously described (42). All experiments were approved by The Ottawa Health Science Network Research Ethics Board (2009009-01H). K562 and Jurkat cells were cultured in RPMI1640 supplemented with 10% fetal bovine serum as described before (43). All cell lines were verified by short tandem repeat analysis and tested for mycoplasma contamination.

### T-ALL xenograft model

Wild-type (WT) control, *−31CBS*-KO or *−31CBS*-INV Jurkat cells were transduced with lentiviral particles expressing the mCHERRY fluorescent protein and luciferase (FUW-CHERRY-puro-LUC) (44). The viability and transduction efficiency of cells was measured by flow cytometry and was equivalent for all conditions. NSG mice (6–8 weeks old, The Jackson Laboratory) were maintained under sterile conditions. Mice were sublethally irradiated at 3-Gy total body irradiation and anesthetized with a solution of 10% ketamine and 5% xylasine. NGS mice were injected with WT control, *−31CBS*-KO or *−31CBS*-INV Jurkat cells at a dose of 50 000 cells per mouse by intrafemoral injection. Leukemia progression for each condition (control, *−31CBS*-KO or *−31CBS*-INV) was then measured 1, 3, 7, 10 and 13 days after injection by *invivo* bio-imaging using the in Vivo Imaging System (IVIS, Xenogen). Briefly, mice were anesthetized and given an intraperitoneal injection of 100 mg/kg D-luciferin (Perkin Elmer #122799). After 5–10 min. luciferase activity was measured by IVIS. Animals were used in accordance with a protocol approved by the Institutional Animal Care and use committees of the Ottawa Hospital Research Institute and the Pennsylvania State University.

### RNA extraction, qRT-PCR and RNA-seq

Total RNA was extracted using TRIzol Reagent (Invitrogen). A total of 1 μg of total RNA was subjected to reverse transcription with M-MLV Reverse Transcriptase (New England Biolabs) and analyzed by the CFX96™ Real-Time System (Bio-Rad). RNA-seq library was prepared by ‘TruSeq Stranded mRNA Library Prep Kit’ (Illumina, #RS-122-2101), and paired-end RNA-seq was performed by the Penn State University Genome Sciences and Bioinformatics Facility using the Hiseq2500 (100PE) platform with 40 M reads per sample according to standard protocols. The primers are listed in the Supplementary Table S1.

### ChIP, ChIP-seq and ATAC-seq

ChIP and ChIP-seq were performed as previously described (30,45–46). Briefly, cells were harvested and fixed with 1% formaldehyde. Chromatin was fragmented using a Bioruptor™ UCD200 sonicator (Diagenode SA, Belgium) on high power at 4°C and precipitated using antibodies against *CTCF* (Millipore, #07-729), *H3K4me3* (Millipore, #04-745), *H3K4me2* (Millipore, 07-030), *H3K27Ac* (Abcam, #ab4729) and *H3K9/14ac* (Diagenode, #C15410005). ChIP-DNA was recovered using phenol/chloroform/isoamyl alcohol and assayed by quantitative polymerase chain reaction (qPCR). ChIP primers are listed in the Supplementary Table S1. For ChIP-seq, the ChIP DNA library was prepared by ‘TruSeq ChIP Sample Preparation Kit’ (Illumina, #IP-202-1024). Assay for transposase-accessible chromatin using sequencing (ATAC-seq) was performed as previously described (30). Briefly, 5 × 10^4^ cells were harvested and lysed in lysis buffer on ice. Nuclei were extracted and processed by ‘Nextera DNA Library Preparation Kit’ (Illumina). Libraries were purified with the Qiagen MinElute PCR Purification Kit. ChIP-seq and ATAC-seq libraries were sequenced using the Hiseq2500 (100PE) platform with 25M reads per sample.

### Sequencing data analysis

RNA-seq, ChIP-seq and ATAC-seq analyses were performed as previously described (30). In brief, sequencing reads were aligned to human genome (hg19) using TopHat (version 2.0) and Bowtie2 (47–49). A detailed data analysis protocol is provided in Supplementary Materials and Methods. Sequence reads have been deposited in the National Center for Biotechnology Information Gene Expression Omnibus (NCBI GEO) under accession number GSE135320.

### Chromosome conformation capture (3C), circular chromosome conformation capture (4C) and Hi-C

Chromosome conformation capture (3C) assay was performed as described previously with minor modifications (40). In brief, 2 × 10^6^ cells were cross-linked with 2% formaldehyde. Nuclei were extracted and digested with 800U of *BamH1* or *DpnII* (New England Biolabs) at 37°C overnight with shaking. The digested chromatin was treated with 400U of T4 DNA ligase (New England Biolabs) to ligate interacting DNA fragments at 16°C for 3 days. The ligated chromatin was reverse-crosslinked, followed by phenol/chloroform extraction to purify 3C DNA. Purified 3C ligated DNA was amplified using PCR, the products were visualized on a 2% agarose gel with SYBR safe (Life Technologies). The PCR products were cloned into pGEM-T Easy vector (Promega) for Sanger sequencing (Genewiz). Relative crosslinking frequencies were calculated and plotted after normalization to the loading control. The 3C primers are listed in the Supplementary Table S1.

The circular chromosome conformation capture (4C) assay was performed as described previously (30,50) with minor modifications. Briefly, 1 × 10^6^ cells were cross-linked with 2% formaldehyde and digested with restriction enzyme overnight. Circular ligated chromatin was reversely cross-linked. Then, 4C DNA library was prepared by ‘TruSeq ChIP Sample Preparation Kit’ (Illumina, #IP-202-1024) for further sequencing.

Hi-C assay was performed to generate a genome-wide interaction as described previously with Arima-HiC Kit (Cat: A410030) (https://arimagenomics.com/) with minor modifications. In brief, 2 × 10^6^ cells were collected and cross-linked in 10 ml of phosphate-buffered saline (PBS) buffer containing 1% formaldehyde at room temperature for 10 min. The reaction was quenched by 0.125 M glycine solution. Cross-linked cell pellet was washed in 1× PBS buffer and collected. Cross-linked cell pellet was treated with lysis Buffer in a tube and incubated at 4°C for 15 min, and then added conditioning solution to incubate at 62°C for 10 min. Add stop solution and incubate at 37°C for 15 min. Cell pellet was digested with reaction buffer and restriction enzyme cocktail to digest chromatin overnight or for at least 2 h at 37°C with rotation. Digested DNA was purified with DNA purification beads (AMPure XP Beads), and then quantified its concentration with Qubit. A total of 750 ng of DNA per sample was sheared through sonication (Bioruptor) with default parameters (30 s ON, 30 s OFF pulse intervals). Fragmented DNA must then be size-selected to have a size distribution between 200 and 600 bp. 250ng of size-selected DNA was used to generate sequence library with KAPA Hyper Prep Kit (Catalog # KK8500, KK4824 and KK8502). Final libraries were submitted to paired-end sequencing of 100 bp length on an Illumina HiSeq 2500. The detailed Hi-C data analysis protocol is provided in the Supplementary Materials and Methods. Sequence reads have been deposited in the National Center for Biotechnology Information Gene Expression Omnibus (NCBI GEO) under accession number GSE135320.

### CRISPR/Cas9 for genome editing

We generated −31*CBS* deletion and inversion cell lines by CRISPR/Cas9 technology. Single-guide RNA (sgRNA) oligos (Sigma) were designed using the CRISPR Design Tool (crispr.mit.edu) according to the target genome sequence and cloned into a pLKO5.sgRNA.EFS.tRFP (Addgene, # 57823) or pL-CRISPR.EFS.GFP (Addgene, # 57818) lentiviral vector. After lentiviral infection, GFP^+^/RFP^+^ cells were sorted by flow cytometry. Single cell clones were then selected, and −31*CBS* deletion and inversion clones were verified by genotyping PCR and Sanger sequencing. The genotyping primers are listed in the Supplementary Table S1.

## RESULTS

### *−31CBS* is critical for *TAL1* expression in erythroid cells, but not in TAL1-expressing T-ALL cells

**Figure 1.**
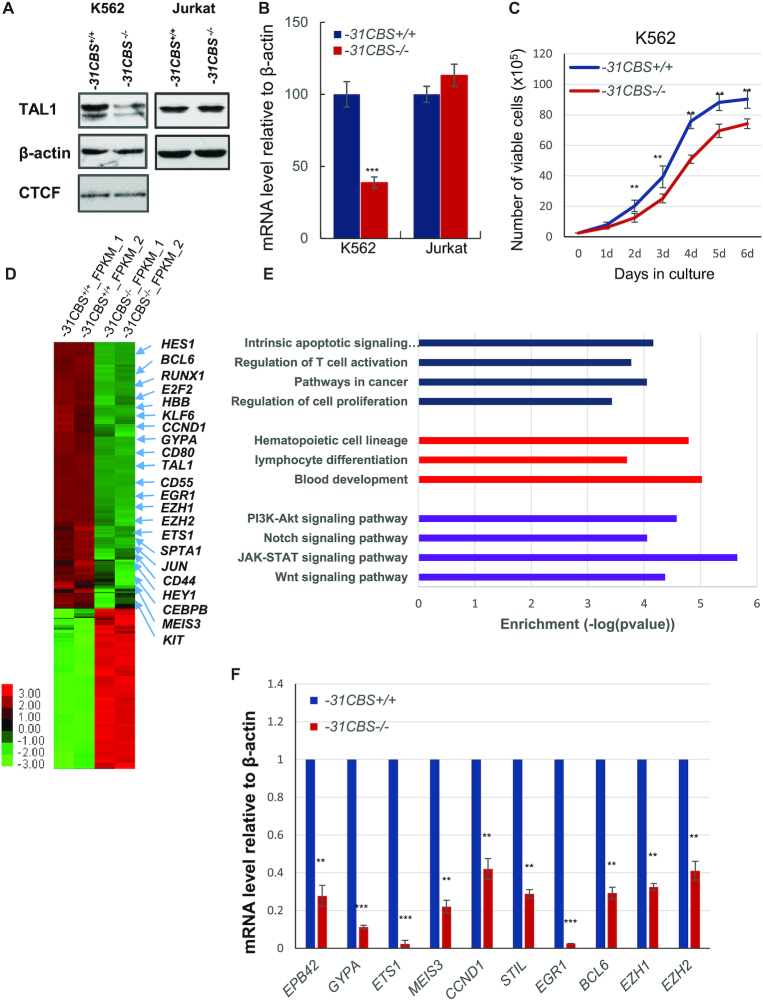
Deletion of *−31CBS* inhibits *TAL1* expression in K562 cells, but not T-ALL cells. (**A**) Western blot analysis of TAL1 protein levels in the *−31CBS* deletion clone in K562 (Clone D10) or Jurkat cells compared to WT control. β-actin is served as a loading control. (**B**) RT-qPCR analysis of *TAL1* mRNA levels in the *−31CBS* deleted K562 (Clone D10) or Jurkat cells compared to WT control. (**C**) Proliferation curves of the WT control and *−31CBS^−/−^* K562 cells (Clone D10) were assessed by counting cell viability for 6 days. (**D**) Heat map of RNA-seq analysis shows upregulated and downregulated genes upon *−31CBS* deletion in K562 cells. (**E**) Downregulated genes upon the *−31CBS* deletion were analyzed and annotated by Gene Ontology analysis. (**F**) RT-qPCR analysis and confirmation of representative downregulated genes identified in the RNA-seq analysis. The data are presented as mean ± SD from three independent experiments, *P < 0.05, **P < 0.01, ***P < 0.001 by student’s *t*-test.

CTCF ChIP-seq study in different hematopoietic cells has established that there are four CBSs flanking *TAL1* gene, *+57CBS*, *+53CBS*, *+40CBS* and *−31CBS* relative to *TAL1* TSS. Computational analysis revealed that orientations of all four CBSs are point toward *TAL1* gene (51). Recently, we and others employed extensive 3C analyses to demonstrate that *−31CBS* functions as a nucleation center and recruits *+53CBS, +57CBS*, *+51* erythroid enhancer and *promoter-I* to form an active chromatin domain in much higher frequencies in erythroid cells than in T-ALL cells (39,40). To characterize the direct role of *−31CBS* in chromatin domain organization and *TAL1* transcription, we employed CRISPR-Cas9 to delete the core CTCF motif of the *−31CBS* in the *TAL1-*expressed erythroleukemia K562 and T-ALL Jurkat cells (Supplementary Figure S1A). Deletion of *−31CBS* (*−31CBS*^−/−^) exhibited very distinct effects on *TAL1* expression between K562 and Jurkat cells (Figure 1A and B). *−31CBS*^−/−^ significantly decreased *TAL1* transcript and protein levels in K562 cells, but not in Jurkat cells (Figure 1A and B; Supplementary Figure S1B and C). *−31CBS^−^^/^^−^* resulted in decreased cell growth of erythroid K562 cells (Figure 1C), while there is no negative growth effect on T-ALL Jurkat cells (data not shown). Given that TAL1 is required for both hematopoietic and leukemia cell survival, we further examined if −*31CBS^−^^/^^−^* affected cell cycle progression of K562 cells. *−31CBS^−^^/^^−^* in K562 cells resulted in increased Sub-G1 apoptotic cells and reduction of the G2 phase (Supplementary Figure S1D).

To explore the mechanism underlying the effect of the *−31CBS^−^^/^^−^*, we performed RNA-seq analysis comparing WT and the two different *−31CBS^−^^/^^−^* K562 cell clones. A total of 1568 genes exhibited more than 2-fold decreases in mRNA levels, whereas 939 genes had increased expression upon *−31CBS* deletion (Figures 1D and Supplementary Table S2). Gene ontology (GO) analysis revealed that pathways involved in hematopoietic cell lineage, lymphocyte differentiation, blood development, T-cell activation, Notch signaling, JAK-STAT signaling and Wnt signaling, etc. were affected by *−31CBS*^−/−^ (Figure 1E). Among them, *TAL1, HBB, GYPA, RUNX1, SPTA1, E2F2, CCND1 and BCL6* were significantly downregulated (Figure 1D). These genes play an important role in hematopoietic/erythroid differentiation and cell cycle regulation. A subset of downregulated genes involved in erythropoiesis and cell cycle were further verified by RT-qPCR analysis (Figure 1F). Perturbation of the cell-cycle pathway by GO term analysis is consistent with the observation that *−31CBS^−^^/^^−^* blocks cell cycle progression leading to apoptosis (Figure 1C and Supplementary Figure S1D).). However, given that −31CBS is overlapped with the *STIL* coding exon, the changes in *STIL* gene expression may be directly resulted from disruption of this exon rather than altered genome organization (Figure 1F).

### *−31CBS* is required for formation of active *TAL1* chromatin domain in erythroid cells

**Figure 2.**
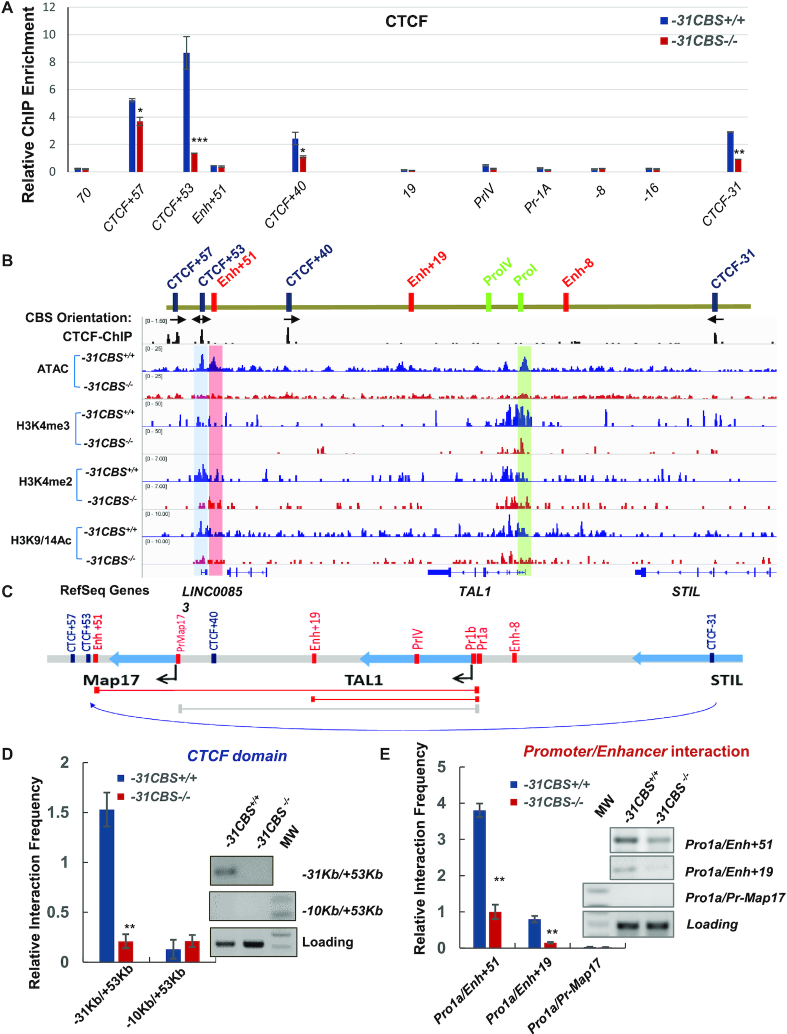
−*31CBS* loss alters CTCF defined chromatin domain and looped enhancer/promoter interaction in the *TAL1* locus in erythroid cells. (**A**) ChIP analysis of CTCF binding across the *TAL1* locus compared the WT control and *−31CBS^−^^/^^−^* cells. (**B**) CTCF binding and CTCF binding site orientations in the *TAL1* locus were indicated. The abundance of ATAC-seq peak as well as enrichment of H3K4me3, H3K4me2 and H3K9/14Ac patterns at the *TAL1* locus analyzed by ATAC-seq and ChIP-seq compared WT control and *−31CBS^−^^/^^−^* K562 cells, respectively. (**C**) Schematic representation of the 3C fragments after restriction digestion in the human *TAL1* locus. Red lines: the primer used to detect known 3C interactions. Grey line: negative control primers. (**D**) PCR analysis of 3C products examining the *−31CBS* mediated DNA looping in the *TAL1* locus compared WT control and *−31CBS^−^^/^^−^* K562 cells. Relative interaction frequency is quantitated in triplicate experiments. (**E**) PCR assay examining the enhancer and promoter interactions in the *TAL1* locus upon *−31CBS* deletion in K562 cells. Relative interaction frequency is quantitated in triplicate experiments. The data from the panel A, D and E are presented as mean ± SD from three independent experiments, *P < 0.05, **P < 0.01, ***P < 0.001 by student’s *t*-test.

It was reported that interaction between *−31CBS* and *+53/+57CBS* facilitates the *+51* enhancer to activate the *TAL1 promoter-I* in erythroid cells (40). Thus, it is likely that *−31CBS^−^^/^^−^* will interfere with chromatin signatures in the *TAL1* locus required for *TAL1* expression. To test this possibility, we performed ChIP to investigate the effect of the *−31CBS*^−/−^ on CTCF binding in the *TAL1* locus. There are four CBSs in the *TAL1* locus located at *−31Kb*, *+40Kb*, *+53Kb* and *+57Kb* relative to the *TAL1* TSS (27,29). While *−31CBS^−^^/^^−^* only reduced CTCF binding to the *−31CBS* in Jurkat cells (data not shown), *−31CBS^−^^/^^−^* in K562 cells significantly inhibited CTCF binding to all CBSs in the *TAL1* locus (Figure 2A). As a control, *−31CBS^−^^/^^−^* did not alter CTCF protein levels in K562 cells (Figure 1A). The data indicate that *−31CBS* may play an important role in organizing the erythroid specific chromatin domain to support *TAL1* transcription in K562 cells.

Next, we examined the effect of *−31CBS*^−/−^ on chromatin accessibility and histone modifications over the *TAL1* locus in K562 cells by ATAC-seq and ChIP-seq analyses. Wild-type cells exhibit highly accessible chromatin regions at the *+53CBS*, *+51* enhancer and *promoter-I* that are correlated with enriched H3K4me2, H3K4me3 and H3K9/K14ac modifications (Figure 2B). *−31CBS^−^^/^^−^* resulted in decreases of enhancer/promoter accessibility and H3K4me3, H3K4me2 and H3K9/K14ac modifications, especially in the +53CBS, *+51* enhancer and the *TAL1 promoter-I* (Figure 2B). The ChIP-qPCR confirmed that *−31CBS^−^^/^^−^* (two clones) leads to diminishment of active chromatin signature in the *TAL1* locus in K562 cells (Supplementary Figures S2A and B). Furthermore, H3K4me3 enrichment was markedly reduced in the promoters of a subset of *TAL1* target genes identified by RNA-seq (Supplementary Figure S2C), perhaps, due to the reduction of *TAL1* expression by *−31CBS* deletion.

Given that CTCF-mediated chromatin organization plays an important role in regulating enhancer/promoter interactions, we carried out 3C analyses to examine whether *−31CBS^−/−^*affects −31CBS loop interactions and *TAL1* enhancer/promoter interaction (Figure 2C). Consistent with previous reports (40), *−31CBS* interacts with *+53 CTCF* site to form an erythroid-specific CTCF loop in WT K562 cells (Figure 2D). As a result of the −31CBS/+53CBS CTCF organization, the *+51* erythroid enhancer also strongly interacts with the *TAL1 promoter-I* to activate *TAL1* gene (Figure 2E). Interestingly, *−31CBS^−/−^* abolished the formation of *+53/−31CBS* chromatin loop (Figure 2D), and significantly reduced the interaction frequencies of the *TAL1 promoter-I* with the *+51* erythroid enhancer or the *+19* enhancer (Figure 2E) demonstrating that *−31CBS* plays a direct role in organizing erythroid genome to allow enhancer/promoter interactions and transcription of *TAL1*.

### The T-ALL-prone *TAL1* promoter-IV differentially interacts with stem cell enhancer in T-ALL cells

**Figure 3.**
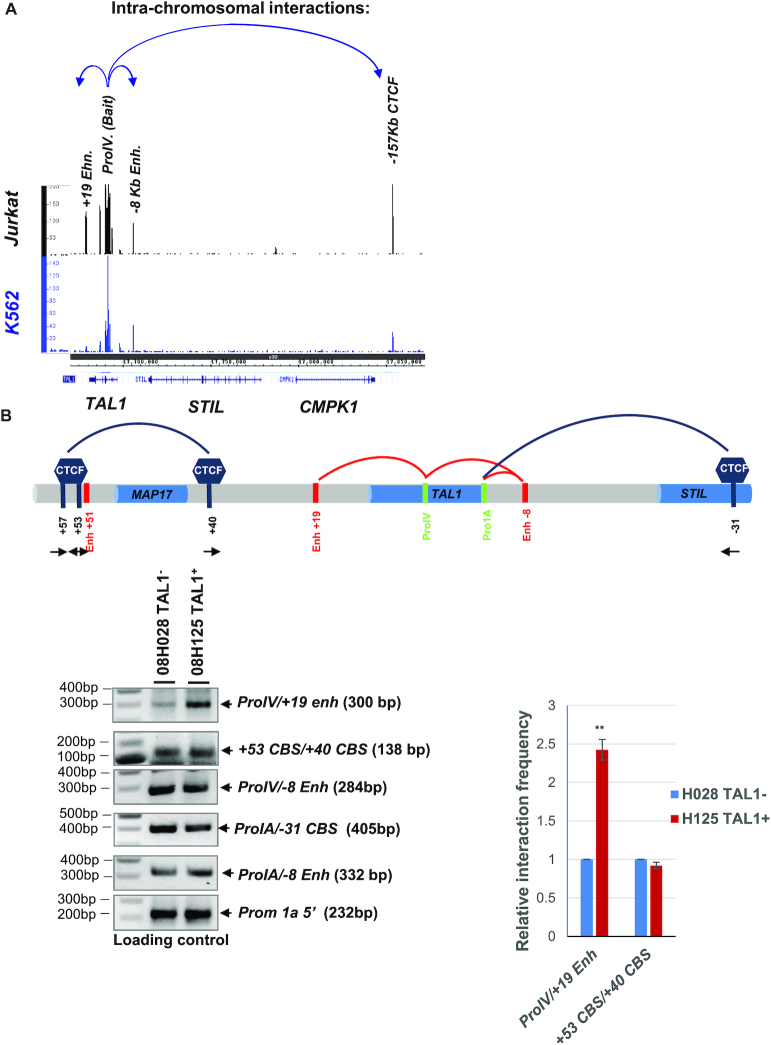
The *TAL1* promoter IV interacts with +19 stem cell enhancer in T-ALL cells. (**A**) Long-range intra-chromosomal interactions in the *TAL1* locus determined by 4C-seq analysis using the TAL1 Promoter IV as a viewpoint compared K562 to Jurkat cells. The blue arrows indicate long-range interactions detected by 4C-seq. (**B**) 3C analysis confirms the interaction between the TAL1 promoter IV and +19 stem cell enhancer in the *TAL1*-negative (08H028) or *TAL1*-positive (08H125) primary T-ALL patient samples. Relative interaction frequency is quantitated from triplicate experiments. The data are presented as mean ± SD, **P < 0.01 by student’s *t*-test.

In addition to the *TAL1 promoter-I*, *promoter-IV* of the *TAL1* gene is also highly active in T-ALL (52,53). Interestingly, all cases of the chromosomal translocation involving the *TAL1* gene are located in the *promoter-IV* region (53–56,53–56). Thus, we reason that the *promoter-IV* may play a differential role in *TAL1* transcription in T-ALL cells. To test this hypothesis, we performed the 4C-seq using the *TAL1 promoter-IV* as a viewpoint, comparing differential long-range interactions associated with the *promoter-IV* of T-ALL Jurkat and erythroid K562 cells. When we compared the cumulative normalized intensities of the *TAL1 promoter-IV* interactome in Jurkat cells with internal control or the interactions in K562 cells, we found that the *TAL1 promoter-IV*-mediated loops were very distinct from that of the *TAL1 promoter-I*. The *TAL1 promoter-IV* interacts with the *+19* stem cell enhancer and the *-8* T-ALL super-enhancer in Jurkat cells, while K562 cells show no interaction between *promoter-IV* and the *+19* enhancer (Figure 3A). It was previously reported that *+40CBS* predominantly interacted with *+53CBS* in T-ALL cell line and patient samples, while *−31CBS* associated with *+53CBS* in erythroid cells (40). This CTCF organization may allow *TAL1* promoter to be activated by enhancers located within or outside the TAL1 locus (40). When we examine the interaction between the *TAL1 promoter-IV* and *+19* enhancer in a TAL1-negative primary T-ALL patient sample (08H028) and a TAL1-positive patient sample (08H125), the *TAL1 promoter-IV* only weakly interacts with the *+19* enhancer in the TAL1-negative patient (08H028) (Figure 3B). In contrast, the *TAL1 promoter-IV* strongly interacts with *+19* enhancer in the TAL1-positive T-ALL patient (08H125) (Figure 3B). Thus, our data reveal that the *+19* stem cell enhancer located 19 Kb downstream of the *TAL1* gene may also drive *TAL1* expression in T-ALL leukemia.

### Inversion of *−31CBS* inhibits *TAL1* expression and TAL1-mediated T-ALL leukemogenesis

**Figure 4.**
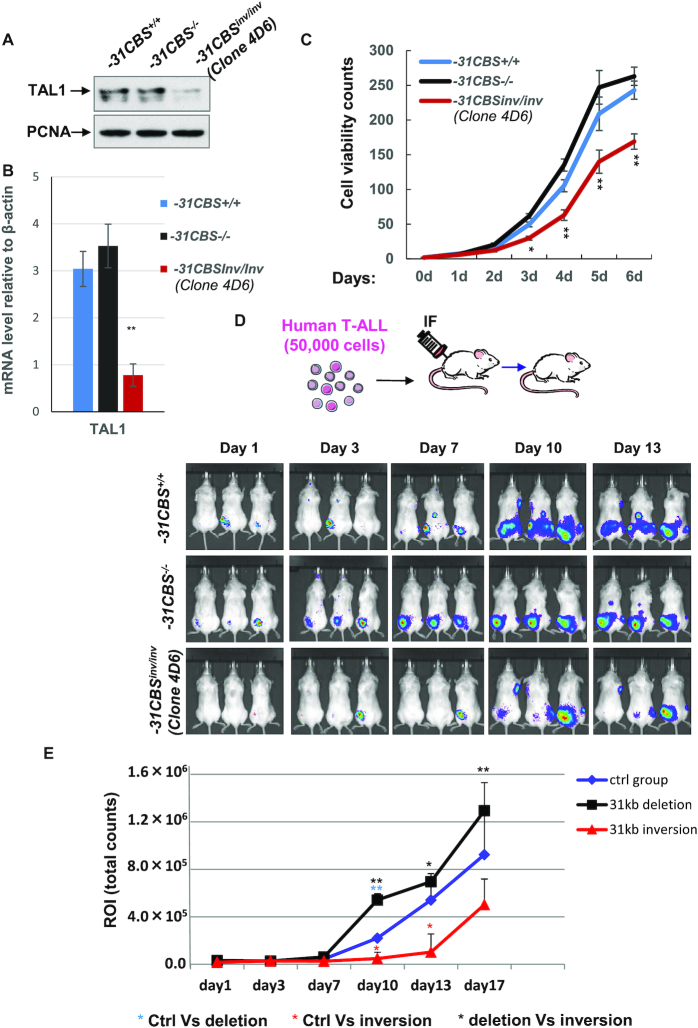
Inversion of *−31CBS* inhibits T-ALL cell proliferation *invitro* and leukemogenesis *invivo*. (**A**) Western blot analysis of TAL1 protein levels in the *−31CBS^−/−^* and *−31CBS^inv/inv^* (Clone 4D6) Jurkat cells as compared to the WT control cells. PCNA serves as a loading control. (**B**) RT-qPCR analysis of TAL1 RNA expression in the *−31CBS^−/−^* and *−31CBS^inv/inv^* (Clone 4D6) Jurkat cells as compared to the WT control. (**C**) Proliferation curves of WT control, *−31CBS^−/−^* and *−31CBS^inv/inv^* (Clone 4D6) Jurkat cells were assessed by counting cell viability. (**D**) A total of 50 000 mCherry-Luc-Jurkat cells are intrafemorally injected into mice. Bioluminescent imaging of tumor burden from mice injected with WT control, *−31CBS^−/−^* and *−31CBS^inv/inv^* mCherry-Luc-Jurkat cells at day 0, 3, 7, 9 or 13. (**E**) Total flux from the respective regions of interest (ROI) (photons/sec) is evaluated in mice injected with WT control, *−31CBS^−/−^* and *−31CBS^inv/inv^* Jurkat cells at day 1, 3, 7, 10, 13 or 17. *P < 0.05, **P < 0.01 by student t test.

It was recently reported that altered *CBS* orientation reshapes the genome organization and leads to changes in CTCF protected gene expression (57,58) suggesting that *CBS* polarity determines the status of gene activity. Convergent orientation of each pair of CBSs flanking *TAL1* gene (Figure 2B) may establish *TAL1* TAD that is required for oncogenic *TAL1* expression. We thus examined whether changing the *−31CBS* orientation would alter genome topology within the CTCF-defined chromatin neighborhood and prevent ectopic activation of *TAL1* in T-ALL cells. Using 2-sgRNA-mediated CRISPR-Cas9 editing, we screened the *−31CBS*-KO Jurkat cell pools for single clones containing *−31CBS* inversion (*−31CBS^inv/inv^*). Two clones that exhibited *−31CBS^inv/inv^* were obtained (Supplementary Figure S3A). *−31CBS^inv/inv^* significantly inhibits *TAL1* transcription and protein levels (Figure 4A and B; Supplementary Figure S3B and C). However, *−31CBS^inv/inv^* does not affect CTCF binding (Supplementary Figure S3D), suggesting that inversion may only alter CBS polarity-mediated genome topology, but not CTCF binding *per se*. Given that TAL1 is required for leukemic cell survival (10–12), we further test if *−31CBS^inv/inv^* affects T-ALL cell proliferation and leukemogenesis *in vitro* and *in vivo* as compared to the vector control and the *−31CBS*^−/−^ clone. *−31CBS^inv/inv^* significantly inhibited proliferation of Jurkat cells (Figure 4C and Supplementary Figure S3E) and colony growth in soft agar (Supplementary Figure S3F), presumably by inhibiting *TAL1* expression. Interestingly, *−31CBS*^−/−^, which slightly increases *TAL1* transcription, indeed enhances cell proliferation (Figure 4C).

To examine whether *−31CBS^inv/inv^* affects T-ALL leukemogenesis *in vivo*, we employed a T-ALL xenograft mouse model (42) by transplanting 50 000 vector control, *−31CBS*^−/−^ and *−31CBS^inv/inv^* Jurkat cells into irradiated NSG mice (Figure 4D, top). All mice transplanted with WT or *−31CBS*^−/−^ Jurkat cells developed leukemia 7 days after transplantation. However, the *−31CBS^inv/inv^* cells showed significant delays and inhibition of T-ALL disease progression (Figure 4D and E). Interestingly, consistent with slightly increased growth of *−31CBS*^−/−^ Jurkat cells *in vitro*, *−31CBS^−^^/^^−^* resulted in more aggressive and faster engraftment of leukemia cells *in vivo* (Figure 4D and E).

### Inversion of *−31CBS* alters *TAL1* genome topology in T-ALL cells

**Figure 5.**
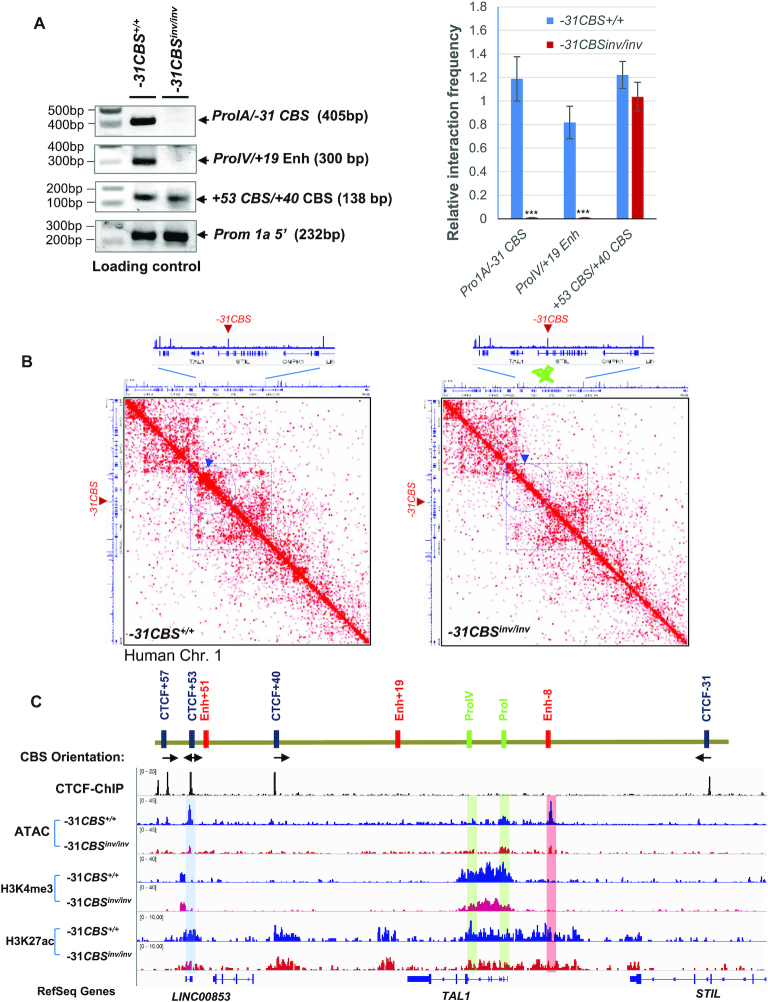
Inversion of *−31CBS* inhibits TAL1 genome topology. (**A**) PCR results of 3C assays examining DNA looping at the *TAL1* locus in WT control and *−31CBS^inv/inv^* (Clone #1–8) Jurkat cells (left). Relative interaction frequency is quantitated in biological triplicate experiments (right). (**B**) Hi-C analysis of genome organization compared between WT control and *−31CBS^inv/inv^* (Clone #1–8) Jurkat cells. (**C**) ATAC-seq and ChIP-seq analysis of chromatin accessibility as well as enrichment of H3K4me3, H3K4me2 and H3K27Ac patterns at the *TAL1* locus compared between WT control and *−31CBS^inv/inv^* (Clone #1–8) Jurkat cells. ***P < 0.001 by student’s *t*-test.

Next, we employed 3C analysis to examine whether *−31CBS^inv/inv^* alters genome topology. Although both *promoter-I* and *promoter-IV* interact with −8 superenhancer in the *TAL1*-negative and *TAL1*-positive T-ALL patient cells (Figure 3B), *−31CBS^inv/inv^* does not prevent the −8 super-enhancer to interact with the *TAL1* promoters (Supplementary Figure S4A). In contrast, inversion resulted in complete loss of interactions of *−31CBS* and the *TAL1 promoter-1a*, and disruption of proximity between the *TAL1 promoter-IV* and *+19* enhancer (Figure 5A and Supplementary Figure S4B). The interactions of the *TAL1 promoter-IV*/*+19* enhancer, as well as the *−31CBS*/*TAL1 promoter 1A* was further validated by sequencing analysis as the fusion molecules constituting two genomic regions were confirmed by Sanger sequencing (Supplementary Figure S4C). We further investigated whether *−31CBS^inv/inv^* alters T-ALL genome organization by Hi-C analysis, comparing vector control and the *−31CBS^inv/inv^* Jurkat cells (Figure 5B, Supplementary Table S3). Inversion of *−31CBS* has not or very little effects on global chromosome organization (Supplementary Figure S5A-S5B), rather it affects chromatin organization at the *TAL1* locus (Figure 5B). In control cells, there is predominant small TAD formed between *+53CBS* and *+40CBS* (blue arrowhead), and a weak TAD between *+53CBS* and *−31CBS* (broken blue cycle dash line) (Figure 5B, left). Interestingly, *+53CBS* also interact with *−157CBS* to form larger TAD (Figure 5B, broken purple square dash line). *−31CBS^inv/inv^* disrupts the TAD formation in the *TAL1* locus, especially the smaller TAD between *+53/57CBS* and *+40CBS* (blue arrowhead), as well as the larger TAD between *+53CBS* and *−157CBS* (broken purple square dash line) encompassing the whole *TAL1* locus (Figure 5B, right). Thus, the data demonstrate that *−31CBS* plays critical role in organizing the 3D *TAL1* genome to support *TAL1* oncogenic transcription program.

### *−31CBS^inv/inv^* remodels chromatin signature leading to perturbation of *TAL1*-driven leukemic transcription program

The CTCF boundary defines the chromatin neighborhood and genome topology. Altering *CBS* may change chromatin modifications and accessibility. To address this, we assessed the effects of *−31CBS^inv/inv^* on enhancer/promoter chromatin accessibility and histone modifications by ATAC-seq and ChIP-seq assays, comparing vector control and *−31CBS^inv/inv^* Jurkat cells. *−31CBS^inv/inv^* leads to decreases in chromatin accessibility in *−31CBS*, *-8* Jurkat super-enhancer, *TAL1 promoter-I* and *+53CBS*. Consistent with loss of chromatin accessibility, H3K4me3, a promoter mark, is significantly decreased in the *TAL1* promoter regions, while enhancer mark H3K27ac is reduced in the *-8* super-enhancer, *promoter-IV*, *+19* enhancer and *+53CBS* (Figure 5C). Effects of *−31CBS^inv/inv^* on histone modifications in the TAL1 locus was further validated in two independent *−31CBS* inverted Jurkat clones (Supplementary Figure S5C and D). This suggests that *−31CBS^inv/inv^* perturbs the CTCF-defined leukemic chromatin neighborhood required for the *TAL1* oncogene activation.

**Figure 6.**
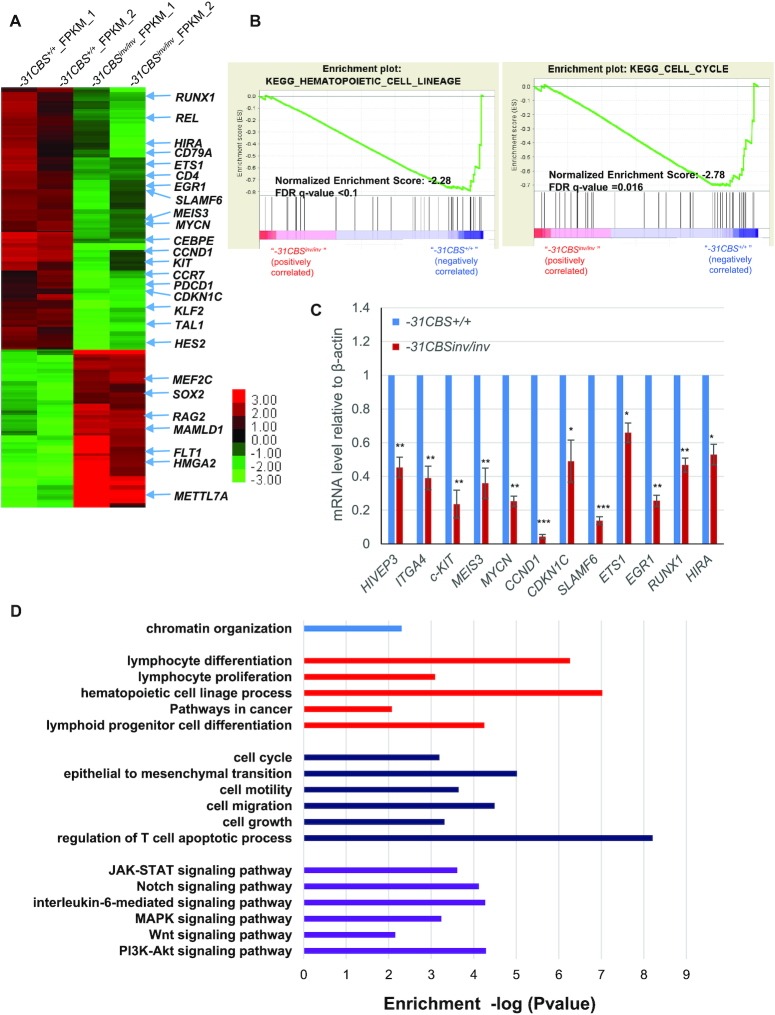
Inversion of *−31CBS* inhibits TAL1-mediated leukemic transcription program. (**A**) Heat map of RNA-seq analysis shows upregulated and downregulated genes upon the *−31CBS* inversion (Clone #1–8) in Jurkat cells as compared to the WT control. (**B**) Enrichment plots by GSEA of regulated target genes involved in cell cycle and hematopoietic cell lineage in the *−31CBS^inv/inv^* (Clone #1–8) Jurkat cells as compared to the WT control. (**C**) RT-qPCR analysis and confirmation of representative downregulated genes identified in the RNA-seq analysis. (**D**) Gene Ontology analysis-identified signaling pathways differently regulated by the WT control and *−31CBS^inv/inv^* (Clone #1–8) Jurkat cells. *P < 0.05, **P < 0.01, ***P < 0.001 by student’s *t*-test.

To investigate whether *−31CBS^inv/inv^* perturbs the *TAL1*-driven leukemic transcription program, we performed RNA-seq analysis comparing the *−31CBS^inv/inv^* Jurkat cells to the vector control. A total of 189 genes exhibited greater than 2-fold decreases in mRNA levels, while 70 genes had increased expression upon *−31CBS^inv/inv^* (Figure 6A and Supplementary Table S4). *−31CBS^inv/inv^* significantly impairs the transcription of many genes important for hematopoiesis, HSC function, cell cycle and leukemogenesis (Figure 6A and B). Many known TAL1 target genes (∼19%) are affected by *−31CBS^inv/inv^* (Supplementary Figure S6A). A subset of known TAL1 target genes important for T-ALL leukemogenesis were confirmed by RT-qPCR (Figure 6C). Together, the data suggest that *−31CBS^inv/inv^* alters the *TAL1*-driven transcription program by remodeling genome topology and chromatin accessibility/modifications. GO analysis further revealed that many of the pathways affected by *−31CBS^inv/inv^* are involved in cell cycle, apoptosis, hematopoiesis, lymphoid progenitor/cell differentiation, JAK-STAT signaling, Notch signaling, IL-6 signaling, Wnt signaling and regulation of cell growth (Figure 6D and Supplementary Figure S6B). These pathways play critical roles in hematopoiesis, lymphoid development and leukemogenesis.

## DISCUSSION

We have identified *−31CBS* as a critical CTCF boundary that differentially regulates the chromatin topology, allowing for differential expression of *TAL1* in erythroid cells and T-ALL cells. However, in *TAL1*-positive T-ALL cells, inversion of *−31CBS* blocks enhancer/promoter communication and alters chromatin signature, including histone modification and chromatin accessibility, leading to inhibition of *TAL1* expression and T-ALL leukemogenesis. Chromatin boundaries, CTCF binding sites in many cases, play a critical role in defining TADs and chromatin signature within the TAD (30,31). CTCF boundary mediated loop formation is proposed to create an insulated neighborhood that organizes genes and enhancers in close proximity to determine a lineage-specific transcription program and to define cell identity (59,60). The chromatin boundary in the genome may not be permanent depending in looped interactions (61). It was proposed that TADs are formed by loop extrusion model (62). In this model, the progressive and dynamic movement of loops driven by cohesion are stalled at chromatin boundaries that required convergent orientation to form TADs by interacting with boundary proteins including CTCF (62). It is particularly interested that RNAs are involved in CTCF-mediated genome organization (63,64). Since that *−31CBS* inversion does not affect the CTCF binding to the *TAL1* locus (Supplementary Figure S3D), it is also possible that changes in convergent boundary orientation alters CTCF association with other boundary factors or lncRNAs leading to altered boundary activity and ability to recruit target genes to its proximity. Nevertheless, change of chromatin insulator polarity or orientation may potentially facilitate or stall the extrusion of loops. Such action may also affect CTCF-driven TAD resulting in disruption of TAD-mediated enhancer/promoter communications and the oncogenic transcription program, such as that of the *TAL1* oncogene in T-ALL, eventually blocking leukemogenesis.

TAL1 is critically required for HSC function and erythroid differentiation (65). Aberrant activation of *TAL1* in T-lymphocytes leads to leukemic transformation in the majority of childhood T-ALL (6). Molecular mechanisms controlling *TAL1* transcription in normal hematopoiesis and leukemic T-cells remains unclear. It is also largely unknown which of TAL1′s enhancers differentially regulate its transcription in normal and malignant hematopoiesis, and how they might do so. We found that *TAL1* expression in erythroid cells is, in part, controlled by a long-range intrachromatin loop that brings the *+51* enhancer into close proximity of the *TAL1 promoter-I*. This interaction is specific to erythroid cells and is absent in T-ALL cells (40). CTCF boundary mediates different chromatin loops of the *TAL1* locus in erythroid and T-ALL cells, thereby providing another layer of regulation to ensure proper *TAL1* expression in distinct cell lineages or differentiation stages (39,40). It is proposed that *−31CBS* plays a crucial role in determining looping interactions at the *TAL1* locus (39). In order to test this notion, we deleted *−31CBS* and showed that *−31CBS^−^^/^^−^* impaired the enhancer and promoter interaction at the *TAL1* locus in erythroid cells, but not affecting *TAL1* transcription in T-ALL cells, suggesting that *−31CBS* may regulate enhancer/promoter interaction in cell context-dependent manner.

However, inversion of *−31CBS* reshapes the genome topology resulting in following consequences (Figure 5). First, disruption of the TAD between *+53/57CBS* and *−157CBS* that protect the whole *TAL1* locus from surrounding chromatin environment. Second, impairment of the association of *−31CBS* with the *TAL1 promoter-I*. Third, decreased the looped interaction between the *+19* enhancer and *TAL1 promoter-IV* (Figure 5). These data support that *−31CBS* acts as a nucleation site crucial in orchestrating looping interactions and defining *TAL1* gene expression. Interestingly, previous studies have shown that the enhancer/promoter loop interactions required looping factor LDB1 and TAL1/GATA/LMO2 transcription super complexes that are present in both normal hematopoiesis and T-ALL leukemia (39,66–68). Thus, it remains to be determined whether and how the TAL1 super transcription complexes including TAL1, GATA factors, LMO2 and LDB1 cooperate with the CTCF boundary to determine enhancer/promoter communication and *TAL1* transcription activity.

The *+19* enhancer in the *TAL1* locus is active in HSCs and progenitor cells, but not in mature erythroid cells (69). Thus, it is not surprising that this enhancer is required for development of hematopoietic stem/progenitor cells, but not necessary for mature hematopoietic lineage (69). Here, we demonstrated that the *TAL1* +19Kb enhancer interacts and activates the *TAL1 promoter-IV* in *TAL1* positive T-ALL cells, but not in K562 erythroid cells (Figure 3). Moreover, the *TAL1* positive T-ALL patient sample exhibited a very strong looped interaction between the *+19Kb* enhancer and the *TAL1 promoter-IV*, while the *TAL1* negative T-ALL exhibits a very weak looped interaction (Figure 3). It is very interesting that the *TAL1 promoter-IV* is specifically active in human T-cell leukemia (53). Thus, our data reveal that the *TAL1 +19* enhancer acts not only as stem cell enhancer, but also as a leukemia specific enhancer to activate the *TAL1* in T-ALL.

Recently, it has been shown that *CBS* orientation determines the genome topology and chromatin looping, which potentially affects promoter function and gene expression (57,58). This may provide a potential strategy to reshape genome organization, especially in the oncogenic loci, to prevent or disrupt the oncogene transcription program for cancer therapy. We reported recently that disruption of a critical CTCF boundary located at the *HOXA* gene locus blocks the oncogenic transcription program and prolongs survival of transplanted AML mice (30). Consistent with this notion, CRISPR-mediated inversion of *−31CBS* disrupts an existing *TAL1* promoter/enhancer interaction and inhibits *TAL1* expression in T-ALL cells leading to interfering leukemia progression. Although *−31CBS* is far away from any known enhancer in the *TAL1* locus, it is likely that change in its orientation alters polarity of boundary and reshapes the TAD organization. In supporting this claim, our Hi-C analysis revealed that altered *−31CBS* orientation disrupts the normal *TAL1* TAD/Sub-TADs, which usually prevents influence signals from the surrounding chromatin environment. It is conceivable that altering CTCF boundary by enhancing or impairment of CTCF binding to boundary can dramatically change TAD structure and oncogenic transcription program to affect oncogenic/leukemogenic processes. Thus, CTCF boundaries that are involved in determining specific topology of leukemic genome and gene expression programs can potentially be targeted against pathogenesis of leukemia.

## DATA AVAILABILITY

Sequence reads have been deposited in the National Center for Biotechnology Information Gene Expression Omnibus (NCBI GEO) under accession number GSE135320.

## Supplementary Material

gkaa098_Supplemental_Files
