# Supplementary material for: Alteration of CTCF-associated chromatin neighborhood inhibits TAL1-driven oncogenic transcription program and leukemogenesis
Source: Nucleic Acids Res. 2020 Feb 22;48(6):3119–33. doi: 10.1093/nar/gkaa098 (PMC7102946; doi:10.1093/nar/gkaa098)
Supplement: gkaa098_Supplemental_Files [file gkaa098_supplemental_files.zip › Supplementary materials.pdf]

Alteration of CTCF associated chromatin neighborhood inhibits *TAL1*-driven oncogenic transcription program and leukemogenesis

Ying Li[1#], Ziwei Liao[2,3#], Huacheng Luo[1], Aissa Benyoucef[4], Yuanyuan Kang[2], Qian Lai[1], Sinisa Dovat[1], Barbara Miller[1], Iouri Chepelev[5,6], Yangqiu Li[3], Keji Zhao[5], Marjorie Brand[4], and Suming Huang[1*]

Genomic data analysis

Sequencing Data Analysis

RNA-seq, ChIP-seq and ATAC-seq analyses were performed as previously described [46]. In brief, sequencing reads were aligned to human genome (hg19) using TopHat (version 2.0) and Bowtie2 [47-49]. Sequence reads have been deposited in the National Center for Biotechnology Information Gene Expression Omnibus (NCBI GEO) under accession number GSE135320.

In RNA-seq, all of sequencing reads were processed and aligned to the human genome assembly (hg19) using TopHat (version 2.0) and Bowtie2 [1-3]. To prevent false positives, a stringent approach was taken to identify differentially expressed genes. First, FPKM (paired-end fragments per kilobase of exon model per million mapped reads) was calculated for each gene and further normalized (RMS-FPKM). To prevent false positives due to the fluctuation of detection among genes with low expression levels, only genes with 50 or more reads in one of the conditions (WT control or -31CBS KO/inversion) were included in the analysis. Differential expression was determined according to abundance estimations (FPKMs) processed with Cufflinks v2.2.1 and Cuffdiff [4]. Differentially expressed genes were identified if the ratio of RMS-FPKM in the two conditions was greater than 2.0 fold, or undetectable in one condition but detectable by more than 50 reads in the other. The scatter plot was based on $\log_2$ transformation of the RMS-FPKM values. Expression level increased or decreased genes were

marked with red or blue, respectively. The GO mapping of differentially expressed genes were performed with Gorilla [5]. The normalized expression data was loaded to Integrated Genomic Viewer (IGV) for comparison.

ChIP-seq raw reads were trimmed with bbduk.sh (https://jgi.doe.gov/data-and-tools/bbtools/bb-tools-user-guide/bbduk-guide/), and aligned to human genome (hg19) using Bowtie2 [2], and the quality of these trimmed data was evaluated by FastQC program [6]. Peaks were identified using MACS2 program and peak annotation with the command "annotatePeaks.pl" from HOMER package [7]. Genome browser tracks were created with the genomeCoverageBed command in BEDTools [8] and normalized such that each value represents the read count per kilobase pair per million mapped (RPKM) and filtered reads, and data tracks of visualization were normalized to the number of fragments falling within all peaks for each sample [9]. BamCoverage was employed to generate the bigWig file of fragment or read coverages, and bamCompare was employed to compare the difference between these two normalized BAM files (e.g. log2ratio) based on the number of mapped reads, including control and experimental datasets [9]. All sequencing tracks were visualized in the IGV genome browsers [10].

Transposase-mediated chromatin accessibility profiling was performed using the Nextera DNA library preparation kit as described previously [11]. In Brief, 5×10^5 cells in single cell suspension were incubated with hyperactive Tn5 transpose to simultaneously cut and ligate adapters for high throughput sequencing at highly accessible chromatin regions. After transposition reaction, the transpose DNAs were purified and subject to PCR using amplification using adapter primers. The PCR products were then subjected to high throughput sequencing on an Illumina NextSeq2500 platform. ATAC-sequencing raw reads were trimmed with bbduk.sh (https://jgi.doe.gov/data-and-tools/bbtools/bb-tools-user-guide/bbduk-guide/), and aligned to human genome (hg19) using Bowtie2 with parameters ("-n 1 -m 1 -p 8")[2], and the quality of

these trimmed data was evaluated by FastQC program [6]. After alignment, SAM files were converted to BAM files and sorted using Samtools [12]. Peak calling was performed using peak calling algorithm MACS2 with parameters ("-g mm -p 1e-9 –nolambda –nomodel –shiftsize=100".) [13], and peak annotation with the command "annotatePeaks.pl" from HOMER package [7] and GREAT [14]. Genome browser tracks were created with the genomeCoverageBed command in BEDTools [8] and normalized such that each value represents the kilobase pair per million mapped (RPKM) and filtered reads, and data tracks of visualization were normalized to the number of fragments falling within all peaks for each sample [9]. BamCoverage was employed to generate the bigWig or bedGraph file of fragment or read coverages, and bamCompare was employed to compare the difference between these two normalized BAM files (e.g. log2ratio) based on the number of mapped reads, including control and experimental datasets [9]. Combined tracks with percentile signal across the cohort were created by quantifying ATAC-seq reads coverage at hg19 assembly of the human genome position with BEDTools coveragegroups [9]. Resulting wiggle files were normalized for sequencing depth by dividing the read counts in each bin by the millions of mapped reads in each sample and were visualized in the IGV genome browsers [10]. DEseq2 (Benjamini-Hochberg adjusted p< 0.1; FoldChange>2; DEseq method) were also performed to find the differential binding sites between two peak files, including control and experimental [15].

For Hi-C data analysis, raw reads were first cleaned to remove adapter and low quality reads with bbmap and bbduk.sh (https://jgi.doe.gov/data-and-tools/bbtools/bb-tools-user-guide/bbduk-guide/). The paired-end sequencing data was trimmed from the 3′ end of enzymatic sequences with homerTools (homerTools trim -3 Arima -mis 0 -matchStart 20 -min 20). Trimmed reads were aligned to human genome (hg19) using Bowtie2 with parameters ("-n 1 -m 1 -p 8") [2]. The remainder of the analysis was performed using juicer [16] and Homer [7]. Paired-end sequencing was used to make a tag directory with makeTagDirectory package from Homer

software. A normalized interaction matrix was generated with analyzeHiC with parameters (1 Mb resolution for all chromosomes, and 100 kb resolution for specific chromosome), and the specific loci interaction of intra-chromatin was also generated with analyzeHiC program in Homer software via parameters (-res 10,000 -superRes 20,000 –pos chromosome location). In depth explanations of normalization, generation of Hi-C correlation matrices, principal component analysis (PCA) and identifying significant interactions were performed as previously described [17]. Hi-C heatmaps were normalized to output the ratio of observed to expected interactions by assuming each region has an equal chance of interacting with other regions in the genome and that regions are expected to interact depending on their linear distance along the chromosome [17]. These interaction matrices for Hi-C heatmap were visualized with Juicebox [18] and Java Treeview.

Table S1. Sequences of primers and probes

Oligonucleotides

Tal1 -31 CBS deletion	sgRNA1	GCAAGAGGATCACTTAGCCCAGG
	sgRNA2	GTATGTTGCCTTCTCGCAAATGG
	US2	TCAACAGCTTGTTTTCCAGGC
	DS2	AGGCCTTTATGATGTTCACAGAGT
	-31 (1)-F	ACCTGATGTACCTGTGTTCTTTCC
	-31 (1)-R	CCCTGTTGGTCCAGTCTGTAAA
RT-PCR primer	Tal1-F	GGATGCCTTCCCTATGTTCA
	Tal1-R	AAGATACGCCGCACAACTTT
	Epb42-F	CTTCACCATCATCCTGTA
	Epb42-R	ACTGGAAATTGGGAATGT
	Gypa-F	ATTGTCAGCAATTGTGAGCATA
	Gypa-R	TGATCACTTGTCTCTGGATTTT
	Ets1-F	GTCGTGGTAAACTCGG
	Ets1-R	CAGCAGGAATGACAGG
	Meis3-F	CCCAGGCTTGGACAGC
	Meis3-R	GCCAGTTCACATTTCTCAAAGA
	Ccnd1-F	GCTGCGAAGTGGAAACCATC
	Ccnd1-R	CCTCCTTCTGCACACATTTGAA
	Stil-F	GAATGCTTCCCTTGTGATGG
	Stil-R	TCAGTTCACAACGGATTGGA
	Erg1-F	CTTCAACCCTCAGGCGGACA
	Erg1-R	GGAAAAGCGGCCAGTATAGGT
	Bcl6-F	CTGATGTTGTCATTGTTG

	Bcl6-R	AACTGGTCTGTAAAGATG
	Ezh1-F	GTGGATGCTACTCGGAAAGG
	Ezh1-R	CCCCACGTACTTGAGAGCAT
	Ezh2-F	AGGGACCAGTTTGTTGGCG
	Ezh2-R	GGGATGACTTGTGTTGGAAAA
	Hivep3-F	CAGTTTTGGTCATCGGAAGTG
	Hivep3-R	GGATGTCAGTATTGCCATTGTATT
	Itga4-F	AGGTGTCCAGCAGAGAAGCTAACT
	Itga4-R	GGATGTCCCGCACATCTTTC
	c-kit-F	TATACAACCCTGGCATTATGTCC
	c-kit-R	TGCGAAGGAGGCTAAACCTA
	Mycn-F	CCACAAGGCCCTCAGTACC
	Mycn-R	TCCTCTTCATCATCTTCATCATCT
	Cdkn1c-F	AGAGATCAGCGCCTGAGAAG
	Cdkn1c-R	GGGCTCTTTGGGCTCTAAAC
	Slamf6-F	TGTTTGTCTTCTGCTTTG
	Slamf6-R	AGTGATGAAGTTGACCTT
	Runx1-F	TCCTAGGCGGTATCATCCTG
	Runx1-R	GTGAAGGCGCCTGGATAGT
	Hira-F	AACAATGAAGCTCCTGAA
	Hira-R	AGGACTGGAGACATATTC
	β-actin-F	GCACAGAGCCTCGCCTTT
	β-actin-R	CGGCGATATCATCATCCAT
ChIP primer on tal1 locus	Prom 1a-F	CTAGCGCCGCTCAACCA
	Prom 1a-R	TGGGCCAAATGATTCATTTTAAT
	Prom IV-F	CGTTTTAAACCCAGTGGCTCTAG
	Prom IV-R	CACGCACACTCTCTCTCACAGAA
	Enh +51-F	GCCTCCTAAGCTTCCTTGATGTC
	Enh +51-R	CAGAAGTGAGACCAATGAGATCGT
	Enh +19-F	TCCAGGAGGGAGTGCCATT
	Enh +19-R	GCCTGCATCCCCCATTG
	Enh -8- F	CCTTGCTTCTATGGGGTACCT
	Enh -8- R	CTCTCATCATTGCCTCCTTCCT
	CBS+57-F	GTGAATGACAGCCATCGTGAT
	CBS+57-R	GGGAGAAGGAGGTGGAGTC
	CBS+53-F	TGGCAGTCCTTCAGTTTCG
	CBS+53-R	CTCTCATCAATCTACGCTTCCTT
	CBS+40-F	TTTTCTCAGGCTAAGCTCTTTGC
	CBS+40-R	GCAAAGTTAGCCAGAGTGTTGTACTC
	CBS-31-F	ACCTGATGTACCTGTGTTCTTTCC
	CBS-31-R	CCCTGTTGGTCCAGTCTGTAAA
	Reg +70-F	GTGGCCACAAAGCAAGGAAT
	Reg +70-R	TCTCTGGAATCTCCAAGGCAA
	Reg -16-F	CAGTAGCAAGCCCAAGTGTAGTAACA
	Reg -16-R	GGAAAGATGCACTAACTGGTCCAT
3C primer	Pro1a	GAGGAGCTGTAGTCGTCAAAT
	ProIV	AGGAGGACGTGGACACCTTCG
	Enh +19	GTCACCCAAGTCATCCTCTCCA
	Enh -8	GGGCTCCAGGGTATGCTAATC
	CBS-31	CTTGAGAGCCTAGGGGAAAC

	CBS+53	TGGGAAGAAATGGCATCTACGC
	CBS+40	GAAACCTGGGAGTCACCTGAA
	Enh +51	GAGTGACCTGACTCGAAC
	Reg -10	CAAATCAGAAGAAAAGACCTGCAA
	MAP17	GCCAAGCAAGGACAGACTTAAGGAGGGTG
	Prom1a 5-F (loading)	ACTGCATTGGCTCCACAAC
	Prom1a 5-R (loading)	CAAGGGCTAGAACAGAAGGGT

Table S2. List of differentially expressed genes comparing WT and *-31CBS$^{-/-}$* K562 cells by RNA-seq.

Table S3. Distribution of Hi-C reads between WT and *-31CBS$^{inv/inv}$*

samples	WT	*-31CBS$^{inv/inv}$*
Total raw read pairs	995,787,635	1,011,266,260
Removed by truncation	32363098(3.25%)	31450381 (3.11%)
Mapping Reads	911245264(91.51%)	914892585(90.47%)
Duplicated reads	25492163(2.56%)	21641097(2.14%)
Self-ligated reads	49590224(4.98%)	46012615(4.55%)
Cis interaction	783222505 (97.44%)	790172733(96.86%)
Trans interaction	20577274 (2.56%)	25615759(3.14%)

Table S4. List of differentially expressed genes comparing WT and *-31CBS$^{inv/inv}$* Jurkat cells by RNA-seq.

Supplementary Figure Legends:

Figure S1. (A) Schematic diagram representing the CRISPR-Cas9 KO of CBS-31 in K562 or Jurkat cells, which is confirmed by PCR-based genotyping and Sanger sequencing. **(B)** Western blot analysis of TAL1 protein levels in the *-31CBS* deletion clone in K562 (Clone #2) cells compared to WT control. β-actin is served as a loading control. **(C)** RT-qPCR analysis of *TAL1* mRNA levels in the *-31CBS* deleted K562 cells (Clone #2) compared to WT control. **(D)** Cell cycle analysis in CBS-31 deletion K562 cells compared with WT control by FACS.

Figure S2. (A) ChIP analysis of the enrichment of H3K4me2/me3 and H3K9/K14Ac across the *TAL1* locus in *-31CBS* deleted K562 cells (Clone D10) compared with WT control cells. **(B)** ChIP analysis of the enrichment of H3K4me2/me3 and H3K9/K14Ac across the TAL1 locus in -31CBS deleted K562 cells (Clone #2) compared with WT control cells. **(C)** ChIP-seq analysis of histone modification H3K4me3 at loci of the TAL1 downregulated representative genes in WT control and *-31CBS* deleted K562 cells.

Figure S3. (A) Schematic diagram representing the CRISPR-Cas9 mediated inversion of *-31CBS* in Jurkat cells, which is confirmed by PCR-based genotyping and Sanger sequencing. **(B)** Western blot analysis of TAL1 protein levels in the *-31CBS*$^{+/+}$ and *-31CBS*$^{inv/inv}$ (Clone #1-8) Jurkat cells as compared to the WT control cells. β-actin serves as a loading control. **(C)** RT-qPCR analysis of TAL1 RNA expression in the *-31CBS*$^{+/+}$ and *-31CBS*$^{inv/inv}$ (Clone #1-8) Jurkat cells as compared to the WT control. **(D)** ChIP analysis of CTCF binding across the TAL1 locus in the *-31CBS* deletion and inversion Jurkat cells as compared to WT control cells. **(E)** Proliferation curves of WT control, *-31CBS*$^{-/-}$ and *-31CBS*$^{inv/inv}$ (Clone #1-8) Jurkat cells were assessed by counting cell viability. **(F)** Soft agar growth of cell colonies comparing among WT, *-31CBS* deleted, and -31CBS inverted Jurkat cells.

Figure S4. (A) PCR results of 3C assays examining changes of DNA looping at the TAL1 locus in WT control and the *-31CBS* inverted Jurkat cells. **(B)** PCR analysis of the *TAL1* promoter IV and +19 enhancer interaction in the *TAL1* locus comparing among TAL1 negative HPB-ALL cells, WT Jurkat cells, *-31CBS* deleted and *-31CBS* inverted Jurkat cells. **(C)** Ligation products of 3C assays in the *TAL1* locus were confirmed by Sanger sequencing.

Figure S5. (A) Hi-C interacting map of human chromosome 1 containing the *TAL1* locus. **(B)** Hi-C interacting map of human chromosome 2. **(C)** ChIP analysis of the enrichment of

H3K4me2/me3 and H3K27Ac across the *TAL1* locus in *-31CBS* inverted Jurkat cells (Clone 4D6) compared with WT control cells. **(D)** ChIP analysis of the enrichment of H3K4me2/me3 and H3K27Ac across the *TAL1* locus in another *-31CBS* inverted Jurkat clone (Clone #1-8) compared with WT control cells.

Figure S6. (A) Overlapping differentially expressed genes upon -31CBS inversion with the previously reported *TAL1* KD Jurkat cells [19] (GSE97514). **(B)** Enrichment plots by GSEA of regulated target genes involved in signaling pathways in *-31CBS* inverted Jurkat cells as compared to WT control.

References:

1. Trapnell C, Roberts A, Goff L, et al. Differential gene and transcript expression analysis of RNA-seq experiments with TopHat and Cufflinks. *Nat Protoc*. 2012;7(3):562-578.
2. Langmead B, Trapnell C, Pop M, Salzberg SL. Ultrafast and memory-efficient alignment of short DNA sequences to the human genome. *Genome Biol*. 2009;10(3):R25.
3. Trapnell C, Pachter L, Salzberg SL. TopHat: discovering splice junctions with RNA-Seq. *Bioinformatics*. 2009;25(9):1105-1111.
4. Trapnell C, Williams BA, Pertea G, et al. Transcript assembly and quantification by RNA-Seq reveals unannotated transcripts and isoform switching during cell differentiation. *Nat Biotechnol*. 2010;28(5):511-515.
5. Eden E, Navon R, Steinfeld I, Lipson D, Yakhini Z. GOrilla: a tool for discovery and visualization of enriched GO terms in ranked gene lists. *BMC bioinformatics*. 2009;10:48.
6. Wingett SW, Andrews S. FastQ Screen: A tool for multi-genome mapping and quality control. *F1000Res*. 2018;7:1338.
7. Heinz S, Benner C, Spann N, et al. Simple combinations of lineage-determining transcription factors prime cis-regulatory elements required for macrophage and B cell identities. *Mol Cell*. 2010;38(4):576-589.
8. Quinlan AR, Hall IM. BEDTools: a flexible suite of utilities for comparing genomic features. *Bioinformatics*. 2010;26(6):841-842.
9. Ramirez F, Dundar F, Diehl S, Gruning BA, Manke T. deepTools: a flexible platform for exploring deep-sequencing data. *Nucleic Acids Res*. 2014;42(Web Server issue):W187-191.
10. Robinson JT, Thorvaldsdottir H, Winckler W, et al. Integrative genomics viewer. *Nat Biotechnol*. 2011;29(1):24-26.
11. Buenrostro JD, Wu B, Chang HY, Greenleaf WJ. ATAC-seq: A Method for Assaying Chromatin Accessibility Genome-Wide. *Curr Protoc Mol Biol*. 2015;109:21 29 21-29.
12. Li H, Handsaker B, Wysoker A, et al. The Sequence Alignment/Map format and SAMtools. *Bioinformatics*. 2009;25(16):2078-2079.

13. Zhang Y, Liu T, Meyer CA, et al. Model-based analysis of ChIP-Seq (MACS). *Genome Biol*. 2008;9(9):R137.
14. McLean CY, Bristor D, Hiller M, et al. GREAT improves functional interpretation of cis-regulatory regions. *Nat Biotechnol*. 2010;28(5):495-501.
15. Ross-Innes CS, Stark R, Teschendorff AE, et al. Differential oestrogen receptor binding is associated with clinical outcome in breast cancer. *Nature*. 2012;481(7381):389-393.
16. Durand NC, Shamim MS, Machol I, et al. Juicer Provides a One-Click System for Analyzing Loop-Resolution Hi-C Experiments. *Cell Syst*. 2016;3(1):95-98.
17. Lin YC, Benner C, Mansson R, et al. Global changes in the nuclear positioning of genes and intra- and interdomain genomic interactions that orchestrate B cell fate. *Nat Immunol*. 2012;13(12):1196-1204.
18. Durand NC, Robinson JT, Shamim MS, et al. Juicebox Provides a Visualization System for Hi-C Contact Maps with Unlimited Zoom. *Cell Syst*. 2016;3(1):99-101.
19. Leong WZ, Tan SH, Ngoc PCT, et al. ARID5B as a critical downstream target of the TAL1 complex that activates the oncogenic transcriptional program and promotes T-cell leukemogenesis. *Genes Dev*. 2017;31(23-24):2343-2360.

Figure S1

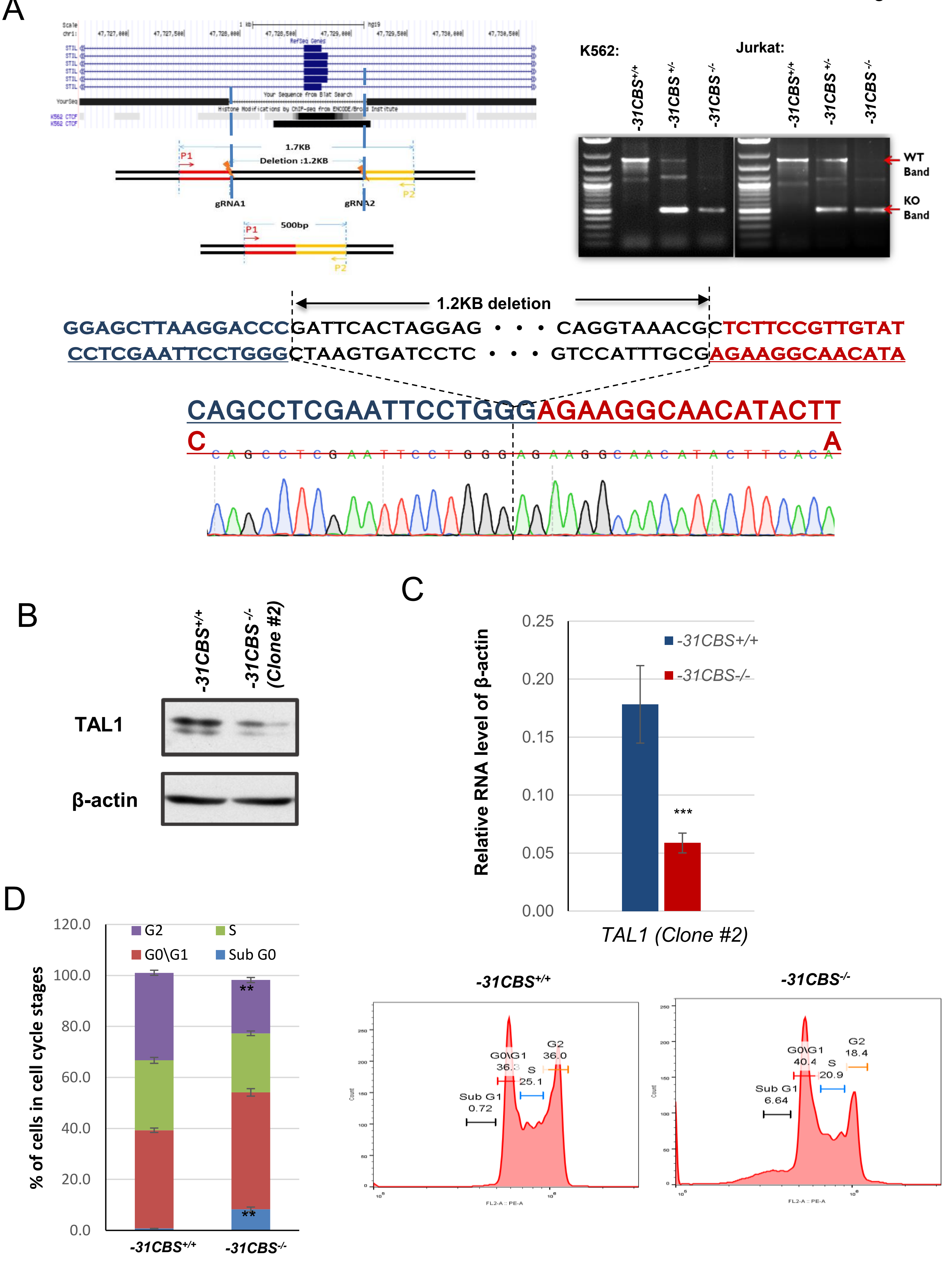
A
1.7KB
Deletion :1.2KB
P1
P2
gRNA1
gRNA2
500bp
K562:
Jurkat:
-31CBS+/+
-31CBS+/-
-31CBS-/-
WT Band
KO Band
1.2KB deletion
GGAGCTTAAGGACCCGATTCACTAGGAG • • • CAGGTAAACGCTCTTCCGTTGTAT
CCTCGAATTCCTGGGCTAAGTGATCCTC • • • GTCCATTTGCGAGAAGGCAACATA
CAGCCTCGAATTCCTGGGAGAAGGCAACATACTT
C
A
B
-31CBS+/+
-31CBS-/- (Clone #2)
TAL1
β-actin
C
Relative RNA level of β-actin
0.25
0.20
0.15
0.10
0.05
0.00
-31CBS+/+
-31CBS-/-
***
TAL1 (Clone #2)
D
% of cells in cell cycle stages
120.0
100.0
80.0
60.0
40.0
20.0
0.0
G2
S
G0\G1
Sub G0
**
**
-31CBS+/+
-31CBS-/-
G0\G1
36.3
G2
36.0
S
25.1
Sub G1
0.72
G0\G1
40.4
G2
18.4
S
20.9
Sub G1
6.64

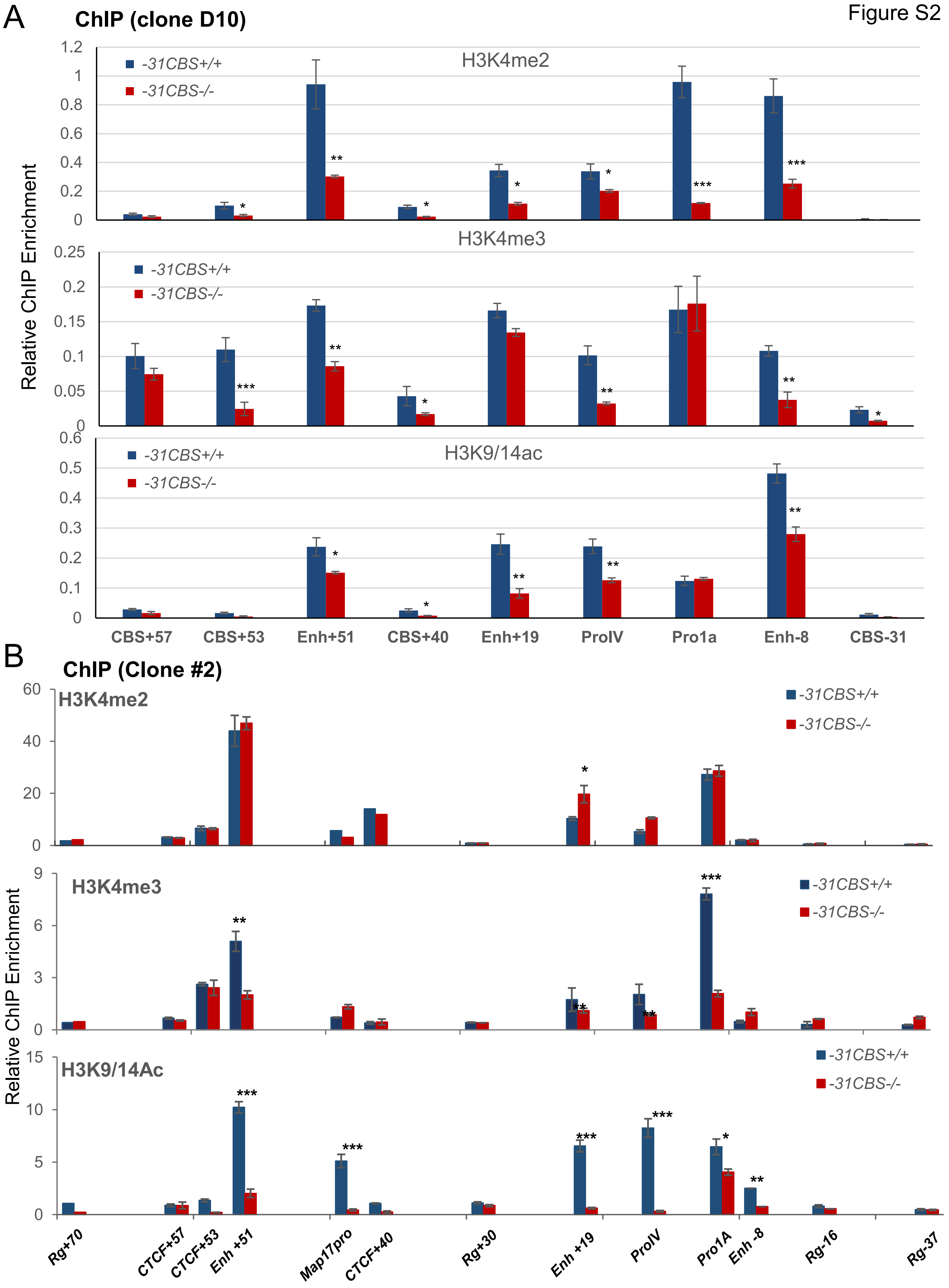
A
ChIP (clone D10)
H3K4me2
H3K4me3
H3K9/14ac
-31CBS+/+
-31CBS-/-
Relative ChIP Enrichment
CBS+57
CBS+53
Enh+51
CBS+40
Enh+19
ProIV
Pro1a
Enh-8
CBS-31
B
ChIP (Clone #2)
H3K4me2
H3K4me3
H3K9/14Ac
Relative ChIP Enrichment
Rg+70
CTCF+57
CTCF+53
Enh +51
Map17pro
CTCF+40
Rg+30
Enh +19
ProIV
Pro1A
Enh -8
Rg-16
Rg-37

Figure S2 cont.

C

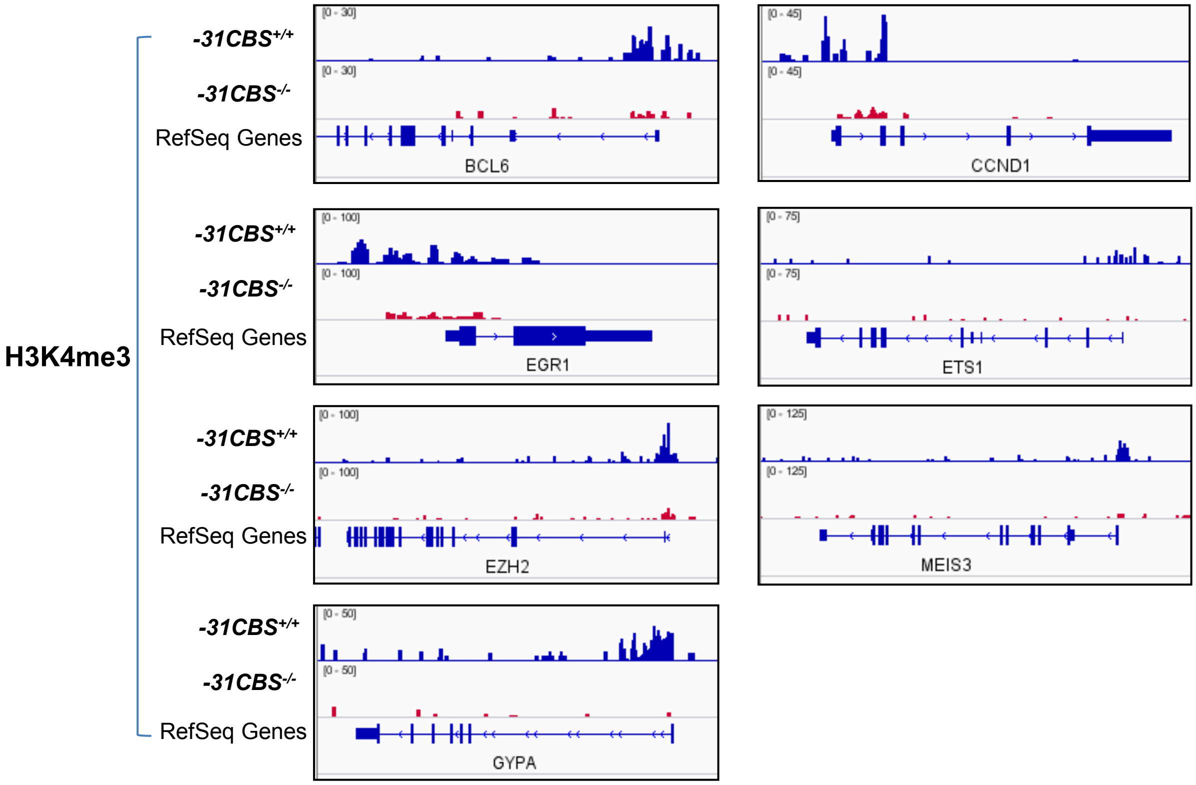

Figure S3

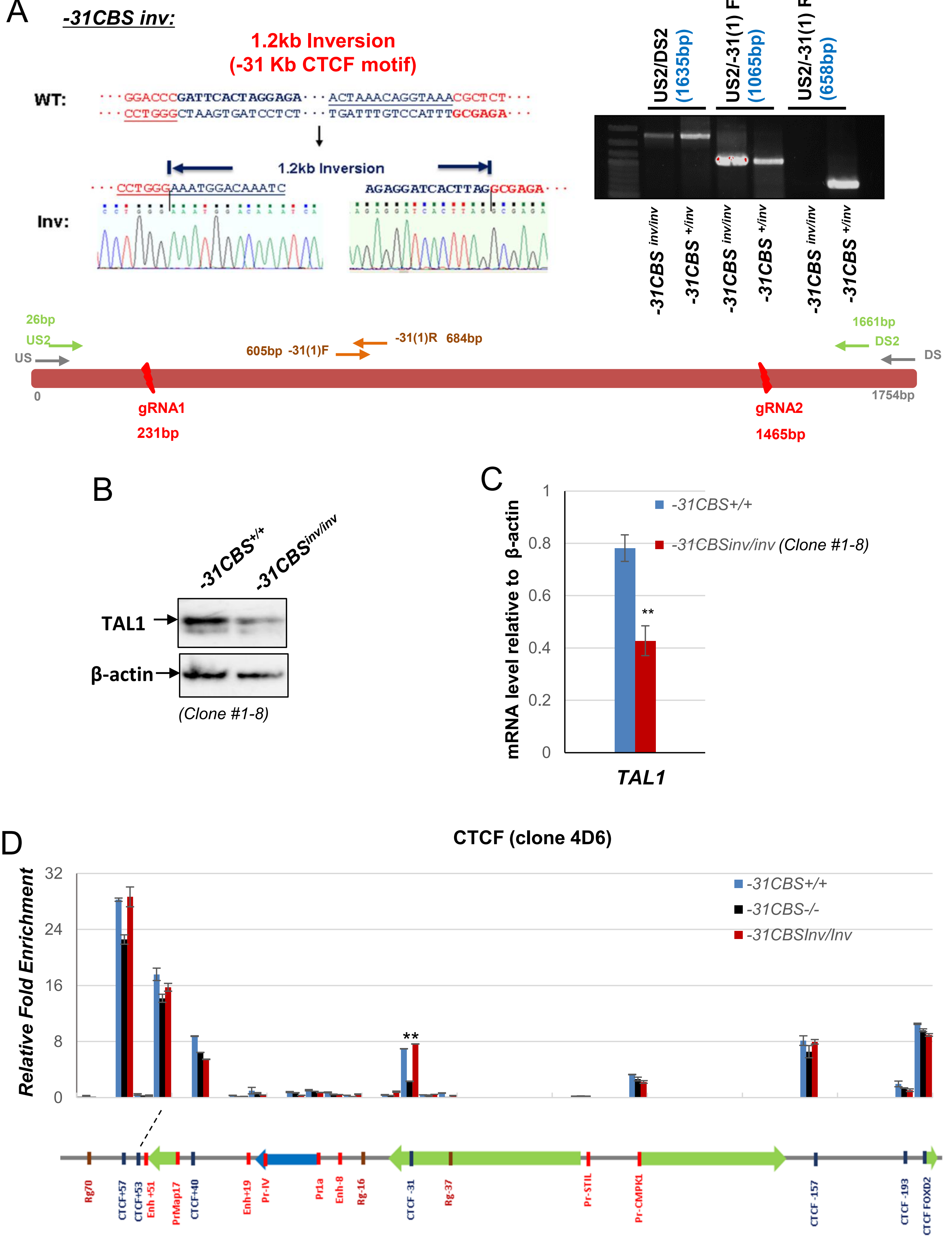
A
-31CBS inv:
1.2kb Inversion
(-31 Kb CTCF motif)
WT:
···GGACCCGATTCACTAGGAGA···ACTAAACAGGTAAACGCTCT···
···CCTGGGCTAAGTGATCCTCT···TGATTTGTCCATTTGCGAGA···
1.2kb Inversion
···CCTGGGAAATGGACAAATC
AGAGGATCACTTAGGCGAGA···
Inv:
US2/DS2 (1635bp)
US2/-31(1) F (1065bp)
US2/-31(1) R (658bp)
-31CBS inv/inv
-31CBS +/inv
-31CBS inv/inv
-31CBS +/inv
-31CBS inv/inv
-31CBS +/inv
26bp
US2
US
605bp -31(1)F
-31(1)R 684bp
1661bp
DS2
DS
0
1754bp
gRNA1
231bp
gRNA2
1465bp
B
-31CBS+/+
-31CBSinv/inv
TAL1
β-actin
(Clone #1-8)
C
mRNA level relative to β-actin
1
0.8
0.6
0.4
0.2
0
-31CBS+/+
-31CBSinv/inv (Clone #1-8)
**
TAL1
D
CTCF (clone 4D6)
Relative Fold Enrichment
32
24
16
8
0
-31CBS+/+
-31CBS-/-
-31CBSInv/Inv
**
Rg70
CTCF+57
CTCF+53
Enh +51
PrMap17
CTCF+40
Enh+19
Pr-IV
Pr1a
Enh-8
Rg-16
CTCF -31
Rg-37
Pr-STIL
Pr-CMPK1
CTCF -157
CTCF -193
CTCF FOXD2

Figure S3 cont.

E

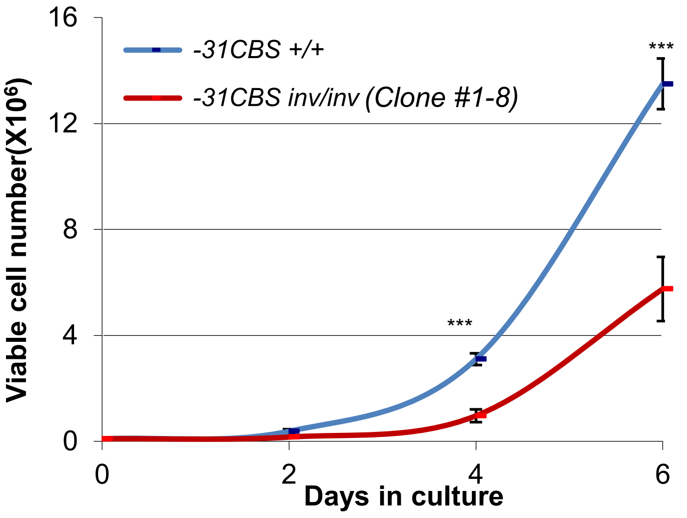

F

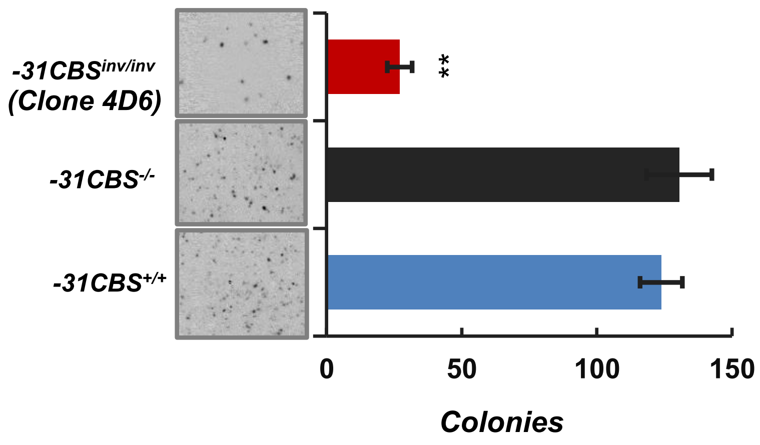

Figure S4

A

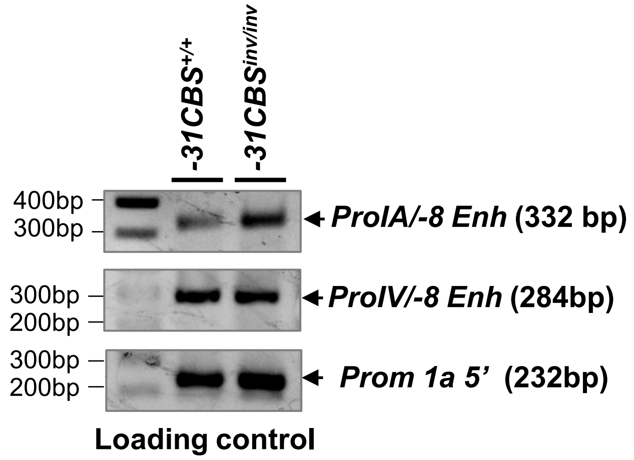

B

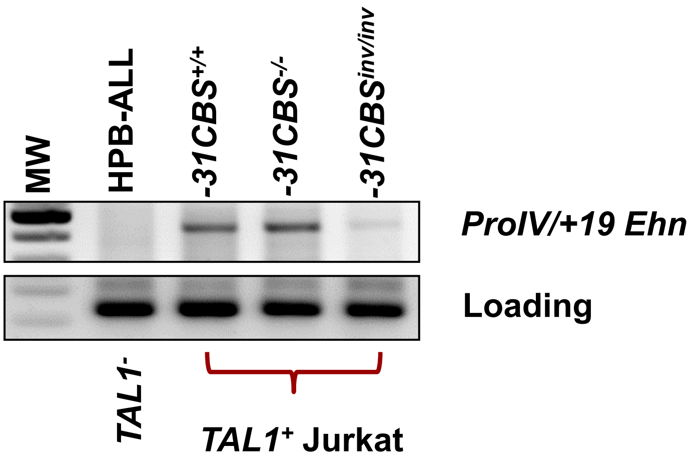

C

ProIA/-31 CBS (405bp)

GAGGAGCTGTAGTCGTCAAATACTCTAGA
ACGATTCAGTCTAAAATCTCCCTCCTCCTT
CATTCTCCCCAAATAAAAACAAACAAAATC
TCTCGGGCGTTCCTTTCTGTAATCCAAATC
AAGTGATGCAGCTTAGTCGCCAACAACCA
TCAGTGTTTGTGAGTGGCTTCTTTGGGGCA
TGGACCTCTGGCTGGTAATCCTAAACCGG
CAGGATTTTCCTAAAATGTGGGGAGGAGC
CGGGAGAGGTCCTCCACA**GATC**ACATTTT
AAGTTTTATAATACTAGTTCCTCCAACCAG
AATTACCCCTTTATTCTGTGTTCTCTTTGCT
TTACACTACTACTCCTCAACAGCCTGTCTT
ATAATTGTTACTAGTCTGCATGTGCTGTT**G**
TTTCCCCTAGGCTCTCAAG

ProIV/+19 Enh (300 bp)

AGGAGGACGTGGACACCTTCGCGGGTGC
AGGCATGTGGCTGGTGTGCTTGGTGGTGG
TGTCGTGGGGGACGGAGAGGAAGGAGTC
CCCTGAGCGTGGGTACCAGGAAAGAGTGT
GCCTGCATGTGCTTTGCTGTGAGCAGAGG
TAGATGTTTGTGGTGTGAAATTCATTCGGA
AACTCAGCGCTAGCTGGACAGGCAAGAGA
TAAG**GATC**TGACTCCAATCCTAGGTAACCT
TAGTCAAGCTGCTTCCCCTCTAGGCCTCAT
TTCCTCCTCCCCACAA**TGGAGAGGATGAC**
TTGGGTGAC

A

Chr1

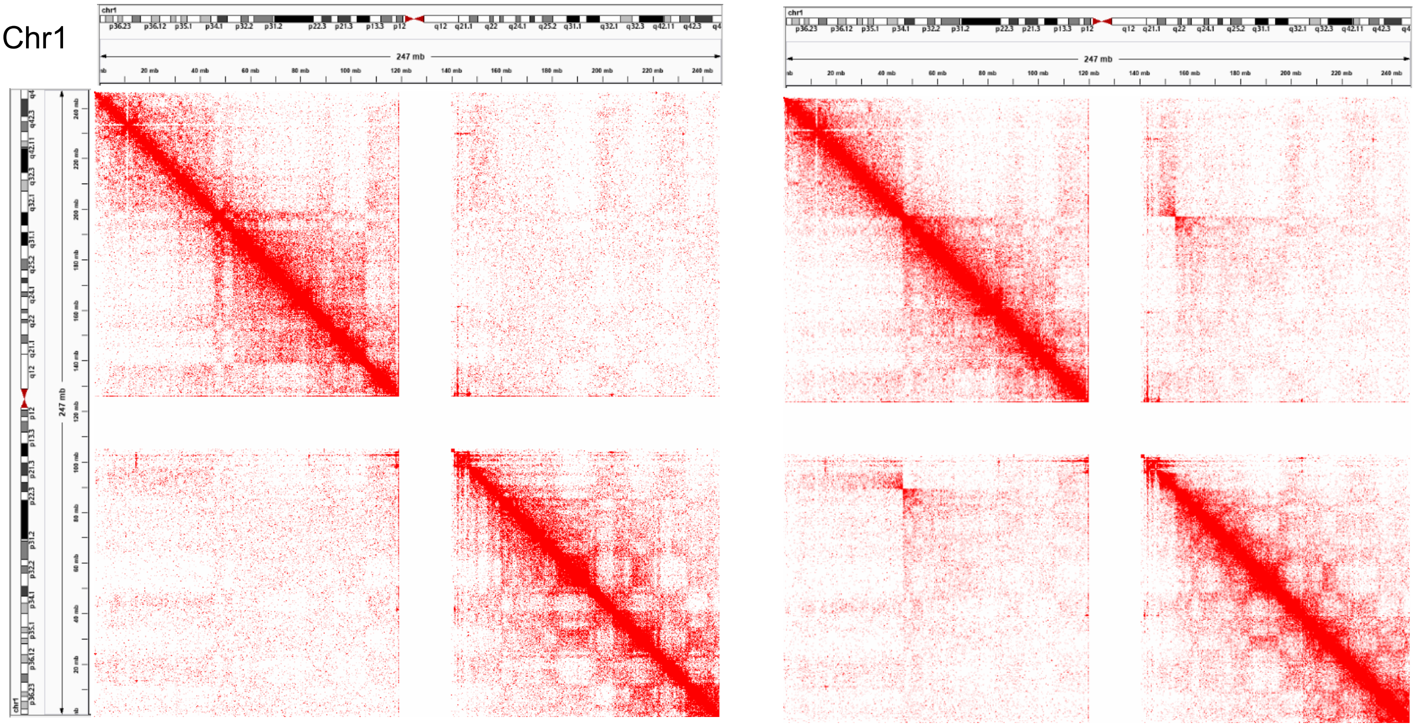

-31CBS$^{+/+}$ *-31CBS*$^{inv/inv}$

B

Chr2

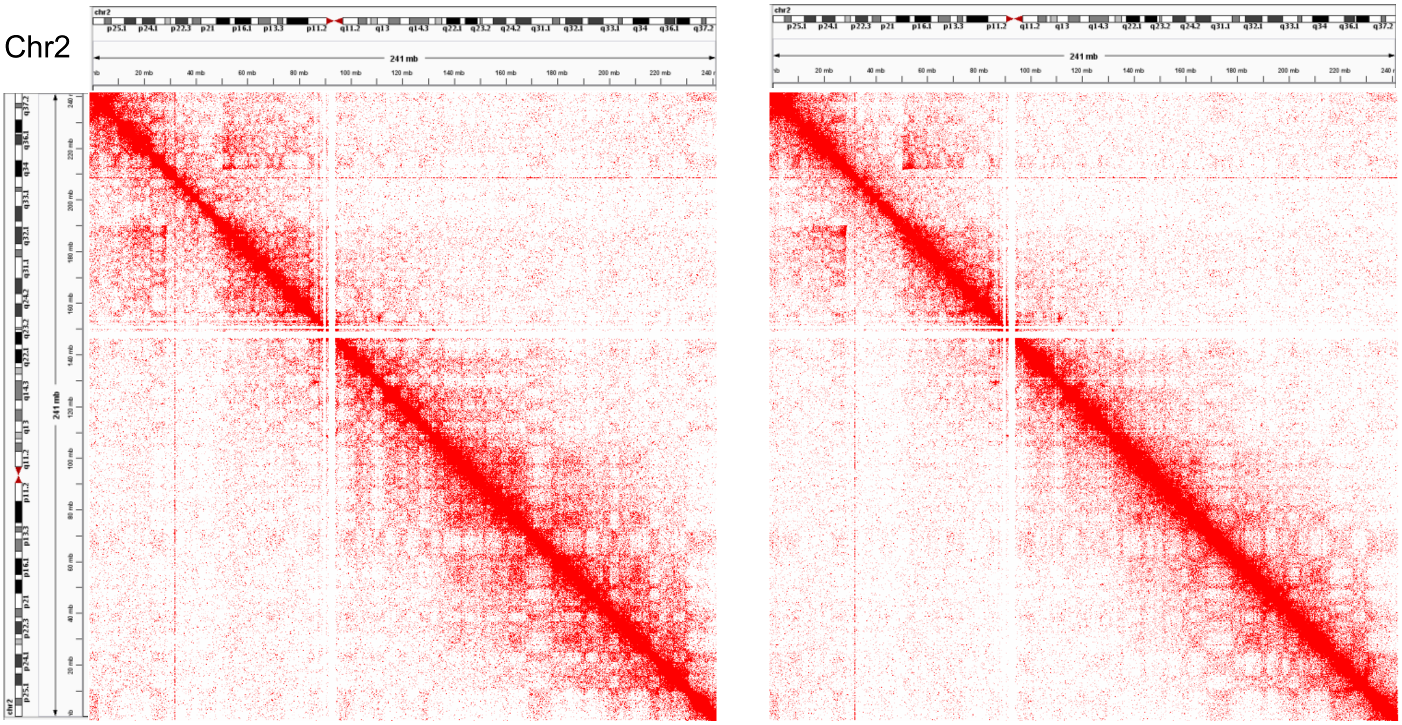

-31CBS$^{+/+}$ *-31CBS*$^{inv/inv}$

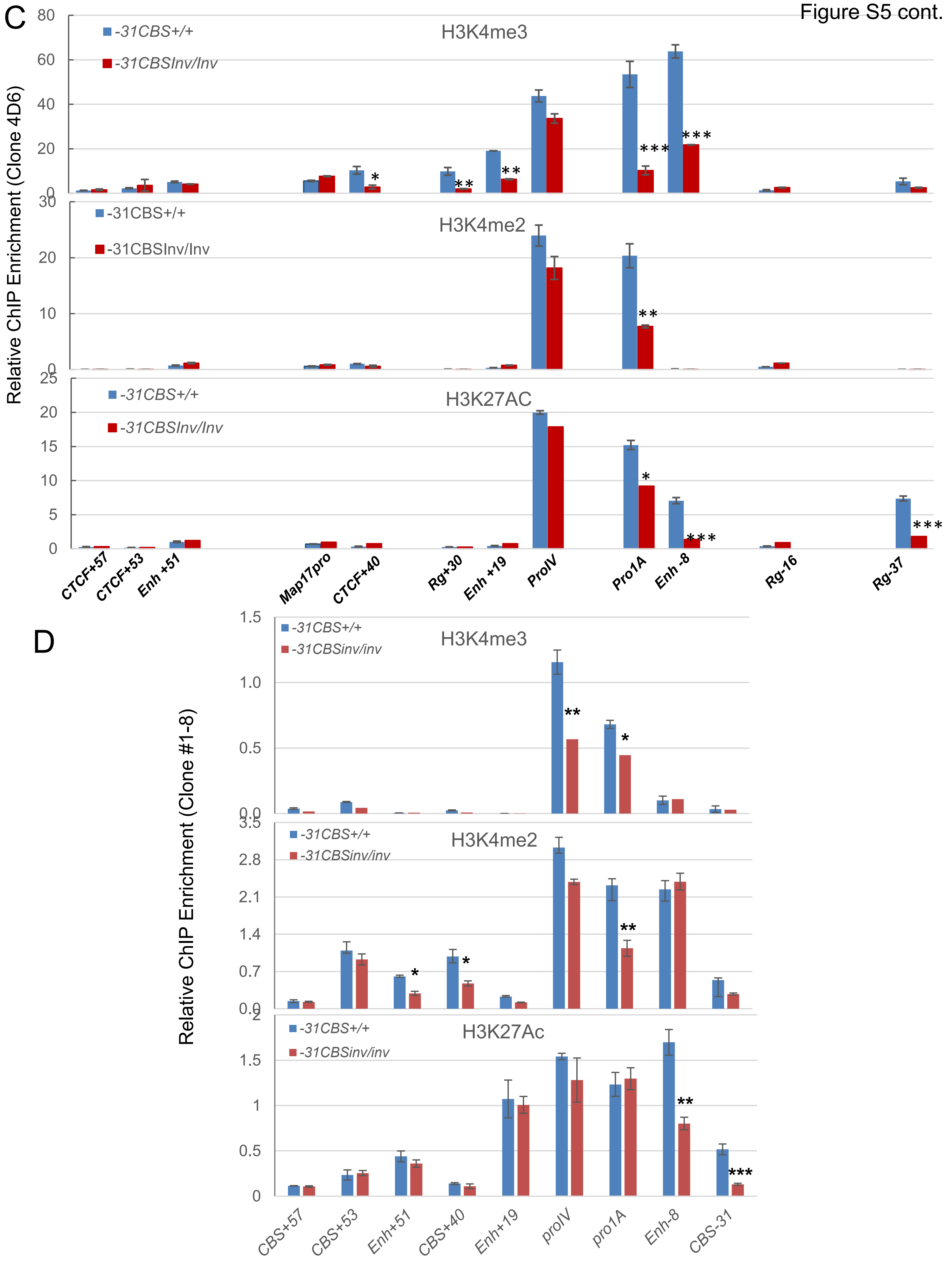
C
Relative ChIP Enrichment (Clone 4D6)
H3K4me3
-31CBS+/+
-31CBSInv/Inv
H3K4me2
-31CBS+/+
-31CBSInv/Inv
H3K27AC
-31CBS+/+
-31CBSInv/Inv
CTCF+57
CTCF+53
Enh +51
Map17pro
CTCF+40
Rg+30
Enh +19
ProIV
Pro1A
Enh -8
Rg-16
Rg-37
D
Relative ChIP Enrichment (Clone #1-8)
H3K4me3
-31CBS+/+
-31CBSinv/inv
H3K4me2
-31CBS+/+
-31CBSinv/inv
H3K27Ac
-31CBS+/+
-31CBSinv/inv
CBS+57
CBS+53
Enh+51
CBS+40
Enh+19
proIV
pro1A
Enh-8
CBS-31

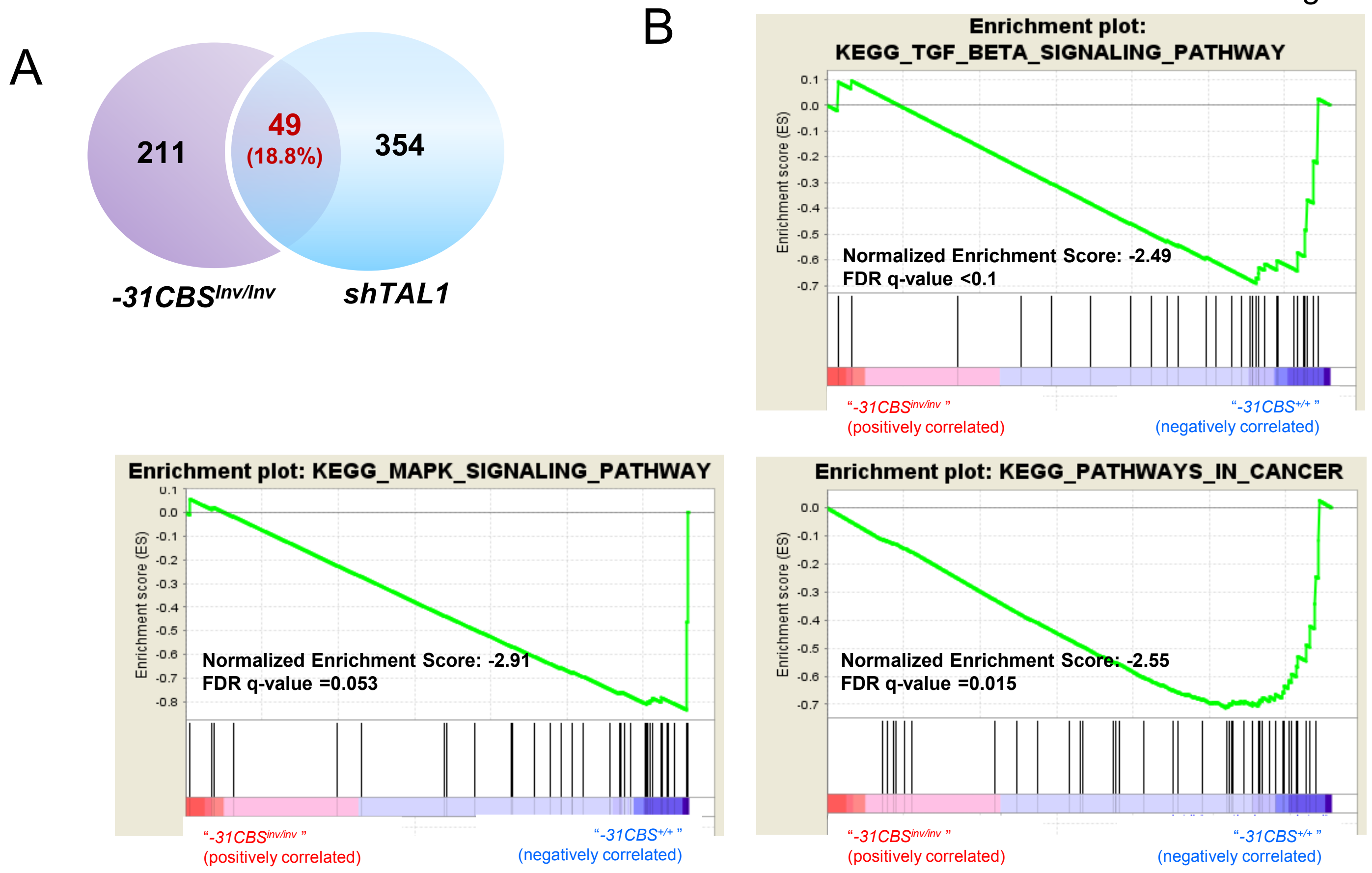
A
211
49
(18.8%)
354
-31CBSInv/Inv
shTAL1
B
Enrichment plot: KEGG_TGF_BETA_SIGNALING_PATHWAY
Enrichment score (ES)
Normalized Enrichment Score: -2.49
FDR q-value <0.1
"-31CBSinv/inv" (positively correlated)
"-31CBS+/+" (negatively correlated)
Enrichment plot: KEGG_MAPK_SIGNALING_PATHWAY
Normalized Enrichment Score: -2.91
FDR q-value =0.053
Enrichment plot: KEGG_PATHWAYS_IN_CANCER
Normalized Enrichment Score: -2.55
FDR q-value =0.015
